# Measuring quality of life in Duchenne muscular dystrophy: a systematic review of the content and structural validity of commonly used instruments

**DOI:** 10.1186/s12955-020-01511-z

**Published:** 2020-08-03

**Authors:** Philip A. Powell, Jill Carlton, Helen Buckley Woods, Paolo Mazzone

**Affiliations:** 1 School of Health and Related Research, University of Sheffield, Regent Court, 30 Regent Street, Sheffield, S1 4DA UK; 2 Department of Economics, University of Sheffield, 9 Mappin Street, Sheffield, S1 4DT UK

**Keywords:** Content validity, Duchenne muscular dystrophy, Patient reported outcome measures, Quality of life, Structural validity

## Abstract

Duchenne muscular dystrophy (DMD) is an inherited X-linked neuromuscular disorder. A number of questionnaires are available to assess quality of life in DMD, but there are concerns about their validity. This systematic review aimed to appraise critically the content and structural validity of quality of life instruments for DMD. Five databases (EMBASE, MEDLINE, CINAHL, PsycINFO, and Cochrane Library) were searched, with supplementary searches in Google Scholar. We included articles with evidence on the content and/or structural validity of quality of life instruments in DMD, and/or instrument development. Evidence was evaluated against the Consensus-based Standards for the selection of health Measurement INstruments (COSMIN) criteria. Fifty five articles featured a questionnaire assessing quality of life in DMD. Forty instruments were extracted and 26 underwent assessment. Forty-one articles contained evidence on content or structural validity (including 37 development papers). Most instruments demonstrated low quality evidence and unsatisfactory or inconsistent validity in DMD, with the majority not featuring direct validation studies in this population. Only KIDSCREEN received an adequate rating for instrument design and a satisfactory result for content validity based on its development, yet, like the majority of PROMs, the measure has not been directly validated for use in DMD. Further research is needed on the validity of quality of life instruments in DMD, including content and structural validity studies in this population.

## Introduction

Duchenne muscular dystrophy (DMD) is an X-linked neuromuscular disorder with an estimated incidence of 1 in 3802–6291 live male births [1, 2]. The disease causes progressive muscle weakness due to an absence of the dystrophin protein, which functions to help keep muscle cells intact. Diagnostic symptoms and functional impairment are evident from as early as two years old and average life expectancy of people with DMD is approximately 25 years [3], although increasingly people with DMD are surviving into their fourth and even fifth decades [4]. The disease progresses through four recognised clinical stages characterised by increased muscle weakness, impaired ambulation and motor functioning, and cardiovascular and respiratory problems [5]. There is no cure for the disease. Current clinical efforts are focused on slowing disease progression and improving the health-related quality of life (QoL) of people with DMD, and health interventions are necessarily evaluated for their cost effectiveness against this objective.

In order to attempt to measure QoL in people with DMD a number of both generic (such as the EQ-5D [6, 7]) and condition-specific (such as the MDCHILD [8]) patient reported outcome measures (PROMs) are used. However, concerns have been raised about the validity of existing PROMs to comprehensively assess QoL in DMD [9]. Given that a number of generic and condition-specific questionnaires are available, researchers and clinicians have to make a critical choice on which measure may be most appropriate for assessing QoL in people with DMD. In order to help inform this decision, evidence-based guidance is needed on the relative validity and psychometric performance of these instruments. There are a number of reviews exploring QoL and associated measures in DMD, with some providing very basic information on their psychometric properties [9, 10]. However, no reviews to date have appropriately evaluated the content validity of available measures when it comes to assessing QoL in DMD. This is a striking omission; content validity has been defined by the COnsensus-based Standards for the selection of health Measurement INstruments (COSMIN) group as the most important property of a PROM [11–13]. Furthermore, prior reviews on QoL in neuromuscular disorders have either not referred to, or used an outdated version of, COSMIN guidance. In the current review we used up-to-date COSMIN methodology to assess the content and structural validity of QoL PROMs in DMD [11].

Content validity refers to the extent that the content of a PROM adequately reflects the target construct that is intended to be measured [14]. It can be subdivided into the judged ‘relevance’, ‘comprehensiveness’, and ‘comprehensibility’ of a PROM, in assessing the construct of interest within a target population and context [13]. ‘Relevance’ of a PROM refers to whether the items are relevant for the construct, target population, and context of use of interest; the response options and recall period of a PROM should also be appropriate and relevant. ‘Comprehensiveness’ is used to describe the extent to which all key aspects of the construct of interest are covered in the PROM. Finally, ‘comprehensibility’ pertains to the understanding of the items and response options by the population of interest [13].

A thorough assessment of a PROM’s content validity should include studies presenting information on content validity in the population of interest, but also consider the initial PROM development paper(s) (i.e. literature describing studies on the development of the PROM) and the content of the PROM itself [12, 13]. The consideration of development studies is important, because the quality of how the PROM was developed (e.g. was there a clear description of the construct to be measured? were patients involved? etc.) necessarily has an impact on the evaluation of the content validity of a PROM in its subsequent use. Thus, COSMIN recommends that the quality of PROM development is rated and assessed prior to the quality of any content validity studies [13]. Furthermore, content validity should form the first step of the assessment of the validity of a PROM, as it is integral to that PROM’s usefulness in doing the job it was designed to do, and influences all other measurement properties [15, 16]. For example, a psychometrically responsive and internally consistent instrument is of little use if it is not measuring what it is intended to measure.

COSMIN guidance states that the second most important form of the validity assessment of a PROM is structural validity [15, 16]. Structural validity describes the extent that scores derived from a measure adequately reflect the dimensionality of the construct being measured [17]. Quality of life is usually defined, and thus measured, as a multidimensional construct. Therefore, PROMs that feature multiple dimensions of QoL should be assessed to check they accurately represent the multidimensional structure of QoL in the population of interest. If PROMs are designed to target a single dimension of QoL, assessments should be undertaken to empirically demonstrate their unidimensional nature in the target population. If such assessments are not undertaken, subsequent interpretation of the data (e.g. through generating dimensional scores) may be inaccurate. For the purposes of this review, we define QoL as a multidimensional construct involving physical (e.g., pain, fatigue), psychological (e.g., mood, self-efficacy), and social (e.g., participation, stigma) components, based on the Comprehensive Model of QoL in Muscular Dystrophy (CMQM) [9], and use this to define the construct of interest. We choose to define QoL as a subjective construct and do not include purely functional performance or assessment scales that may impact on QoL. In this review, we consider multi-item PROMs that assess *at least* one aspect of QoL in people with DMD.

When evaluating a PROM, content and structural validity can be meaningfully assessed against up-to-date published standards by the COSMIN group, derived from international expert consensus [12, 14]. These ratings incorporate actual evidence on PROM validity and the quality of that evidence. For example, regarding a PROM’s ‘comprehensiveness’, a positive rating can be given based on a content validity study if: (i) the study quality was not rated as inadequate; (ii) patients or professionals were interviewed; and (iii) no key concepts were missing. For structural validity, a positive rating is given if good model fit is observed in CFA or in IRT/Rasch (see Methods), and can be appraised alongside a rating for the study’s quality. The full COSMIN standards and methodology for assessing PROMs are comprehensive and available in accompanying guidance manuals [13, 16], which were adhered to when conducting the current review.

This systematic review has been designed to evaluate the content and structural validity of QoL measures used in people with DMD using updated COSMIN guidance [13, 16], to provide researchers and clinicians with a robust evidence-base to help them when selecting PROMs to measure QoL in the Duchenne population. The review makes a unique contribution to the literature in being the first to assess the content validity of PROMs used in DMD and to apply an up-to-date and thorough COSMIN assessment of these measures. There are two main questions being addressed:

1. Which PROMs have been used to assess QoL in published research with boys and men diagnosed with DMD?
2. What is the content and structural validity of these PROMs for use in assessing QoL in boys and men with DMD?

## Methods

The review protocol was registered on PROSPERO [18]. This systematic review has been reported according to the Preferred Reporting Items for Systematic Reviews and Meta-Analyses (PRISMA) checklist [19].

### Search strategy and selection criteria

This review contains two searches. The first search (Search A) was designed to identify PROMs used to measure QoL in DMD in peer-reviewed publications. The second search (Search B) was used to identify literature reporting on the measurement properties of these PROMs in DMD. Search B also included the recommended practice of searching for the development papers of PROMs to enable a full COSMIN assessment of their content validity [12, 13, 15]. Full copies of the searches are contained in Additional file 1, for reproducibility.

#### Search A and selection criteria

Search A was conducted on 11th April 2018, searching EMBASE, MEDLINE, CINAHL, PsycINFO, and the Cochrane library, from inception. No restrictions on date or language were applied to the search. Search A terms are illustrated in full in Additional file 1 and included: (I) Duchenne muscular dystrophy (and Duchenne*) AND ((II) a search filter provided by the PROM group at the University of Oxford to identify PROMs (available online [20] and in Additional file 1) OR (III) PROMs known to be used in people with DMD based on a prior rapid review of the literature [21].

The following selection criteria were applied to the results of Search A by two independent reviewers: (I) published in English as a full-text original research article (i.e. not including abstracts, editorials, or reviews); (II) used a self-reported, multi-item PROM to assess *at least one* aspect of QoL in males diagnosed with DMD (assisted or proxy-reported versions of PROMs were considered for inclusion so long as a self-report version of that PROM exists); and (III) in case of studies involving mixed clinical samples, at least 75% of the sample (or subgroup), on which data from the PROM was reported, was male diagnosed with DMD. The inclusion criteria were first applied to titles and abstracts of the hits from Search A. Records were selected for full-text review if they matched the selection criteria, potentially matched the criteria, or if doubt existed. Any discrepancy was resolved by a third reviewer. Full text articles were then screened for selection using the selection criteria by two independent reviewers. Any disagreements were resolved by a third reviewer through discussion. Finally, the PROMs themselves identified in the articles were reviewed by two independent reviewers to ensure they met the requisite inclusion criteria (i.e. assessing an aspect of QoL).

#### Search B and selection criteria

Search B was conducted on 18th September 2018, with initial searches on EMBASE, MEDLINE, CINAHL, PsycINFO, and the Cochrane library, from inception. No restrictions on date or language were applied to the search. Search B terms are illustrated in full in Additional file 1 and included: term (I) from Search A AND ((II) PROMs identified in Search A OR term (III) from Search A) AND (IV) a search filter^1^ by the COSMIN group for identifying studies on measurement properties [22] (available online [23] and in Additional file 1). Over and above that of Search A, the following additional selection criteria was applied to the results of Search B: (IV) described data on the content and/or structural validity of the PROMs identified in Search A in males diagnosed with DMD; (V) included a PROM validated in English, with a free/review copy available to access.

As recommended in the COSMIN approach [15], follow up searches were conducted on Google Scholar to identify key development papers for the PROMs identified in Search A and taken forward for review (see Section 3.2). Google Scholar was searched (last searched 14th November 2018) with the names and acronyms of the PROMs (version numbers omitted) and the first 100 hits were screened for inclusion [15]. Search results were initially screened by title, with any relevant and potentially relevant papers exported to a database. Following the removal of duplicates against the primary searches, records were screened by abstract and then full text against selection criteria. As per COSMIN guidance, development papers for the PROMs were not subject to any of the inclusion criteria noted above and were included in any published form [13, 15]. Results of the searches were screened for inclusion by two reviewers.

Finally, citation tracking of all eligible articles identified in Search B was conducted by reviewing references and citations on Google Scholar (last searched 6th February 2019) for any articles not identified in the initial searches that may meet the inclusion criteria. All references and citations were reviewed, except where citations became unmanageable (i.e. > 500 citations), when “Duchenne” was searched for within the citing articles to filter the hits for manageable review. Search results were initially screened by title, with any relevant and potentially relevant papers exported to a database. Following the removal of duplicates against the primary searches, records were screened by abstract and then full text against selection criteria. Results of the searches were screened for inclusion by two reviewers.

### Data extraction and COSMIN risk of bias assessment

Data extraction was undertaken by two reviewers using a pre-prepared data extraction sheet, with consensus on any ambiguities reached through discussion. The data extraction sheet was informed by the tools developed by COSMIN on reporting guidance [16], and included study characteristics (authors, year, journal, country, language, study type); details of the PROM used (name, mode of administration, recall period, total *N* subdomains, subdomain names, total and subdomain *N* items, total and subdomain response levels, total and subdomain score ranges); DMD sample characteristics if applicable (N, age, percentage ambulatory, total and subdomain PROM score, total and subdomain observed ranges); details of PROM development if applicable (construct definition, target population, original language, intended context of use, patient involvement); details of content validity results if applicable (summarised results, e.g. findings from a cognitive debriefing exercise); and details of structural validity results if applicable (analytic model, summarised results, e.g. fit statistics, tests of model assumptions for IRT/Rasch).

The methodological quality of the PROM development papers, and studies on content and structural validity were assessed (at the study level) using up-to-date COSMIN standards via the new COSMIN risk of bias checklist [24]. A total rating for relevance, comprehensiveness, and comprehensibility (content validity aspects) of a PROM is determined separately, alongside a total rating for the methodological quality of a structural validity study [24]. When rating the methodological quality of the studies, each COSMIN standard (or item) is ranked on a 4-point scale: “very good”, “adequate”, “doubtful”, and “inadequate”. Total ratings are determined using the lowest rating for any item for that study (i.e. worst score counts) [25]. Studies were initially rated independently by two reviewers, and, in the case of divergence, consensus was reached in a subsequent face-to-face meeting. This information on risk of bias is used to inform quality of evidence (see section 2.2).

### Assessment of measurement properties

In order to synthesise and assess evidence on content validity, two reviewers independently rated the results of PROM development studies, content validity studies, and the content of the PROM itself on 10 COSMIN criteria [13], agreed upon by international consensus [12]. These criteria included: whether the included items were relevant for (I) the construct of interest, (II) the population of interest, and (III) the context of use of interest; whether the (IV) response options and (V) recall period were appropriate; whether (VI) all key concepts were included; whether (VII) the PROM instructions and (VIII) PROM items and response options were understood by the population of interest as intended; whether (IX) the PROM items were appropriately worded; and whether (X) the response options matched the question. Ratings for each source of evidence were made separately, using COSMIN guidance [13] (p.54) and could either be positive (+), negative (−), or indeterminate (?). Reviewers’ ratings were made based on the judgement of the researchers, who have experience in PROM design and work with people with DMD, including direct qualitative research [26, 27]. When reviewers considered whether the items were relevant or comprehensive for the construct of interest, they were compared against the CMQM [9]. Accordingly, a PROM would be sufficiently comprehensive (+) if it included items covering physical, psychological, and social aspects of QoL. When judging the appropriateness of the recall period, reviewers considered any defined recall period of up to 4 weeks as appropriate (+), as children aged 8 years and above can recall up to this length of time with sufficient accuracy [28]. When rating the appropriateness of response options, bearing in mind the target sample (i.e. a child or adult PROM), reviewers took into account the numerical range, how the response options were visually displayed, and the perceived cognitive complexity of the options (including wordiness, degree of variation throughout the questionnaire, and the use of reversed ordering).

Following the above assessment, an overall (qualitatively synthesised) judgment on the relevance, comprehensiveness, and comprehensibility of each PROM was made, which could be sufficient (+), insufficient (−), or inconsistent (±), using COSMIN guidance [13] (p.58). For example, if all sources of evidence were rated positive (+) for relevance, then the overall rating for the PROM would be sufficient (+). As recommended by COSMIN [13], more weight was given to content validity studies, then development studies, then ratings of the PROM by reviewers. Ratings were compared and combined across the two reviewers by consensus. As per COSMIN guidance [13], only available evidence was taken into account when assessing content validity, so, for example, if there were no content validity studies in DMD available for that PROM, assessment was made based on the ratings of any PROM development studies and the ratings of reviewers. The fact that the PROM had no content validation studies in DMD is then reflected in a lower quality of evidence rating (see below). An example content validity rating spreadsheet for the KIDSCREEN-52, including the rules for synthesising the individual ratings is included in Additional file 2.

Evidence on structural validity was assessed against the updated COSMIN criteria for good measurement properties, using the same rating scale as above [16]. Specifically, a positive (+) rating would be given for a CFA featuring a CFI, TLI or comparable measure > 0.95 OR RMESA < 0.06 OR SRMR < 0.08. For an IRT/Rasch model, a positive (+) rating would be given for no violation of unidimensionally (e.g. assessed with the fit statistics above) AND no violation of local independence (e.g. residual correlations among items after controlling for the dominant factor < 0.20) AND no violation of monotonicity (e.g. evidenced graphically or item scalability > 0.30) AND adequate model fit (e.g. χ^2^ < 0.01, infit/outfit mean squares ≥0.5 and ≤ 1.5 OR Z-standardized values > − 2 and < 2. A negative (−) rating would be given if these criteria were not met in the data and an indeterminate (?) rating would be given if model fit was not reported.

Finally, the quality of the evidence was graded using a modified GRADE approach [29], as either “high”, “moderate”, “low”, or “very low”. The GRADE approach takes into account the risk of bias of studies (or study quality); (in) consistency across studies; imprecision (based on sample sizes); and indirectness (of evidence) [16]. The evidence is assumed to be high, then is downgraded by 1–3 points based on the degree of risk of bias (including quality and absence of content validity studies), 1–2 points based on inconsistency, and 1–2 points based on indirectness. Further details on how to apply all of the above criteria are provided elsewhere in comprehensive manuals, which were followed when conducting this review [13, 16]. The quality of this systematic review itself was appraised against a recently developed COSMIN checklist to assess the quality of systematic reviews of health-related PROMs [22].

## Results

### Results of search A – PROMs used to measure quality of life in DMD

After removing duplicates, 1733 records were identified through database searching for Search A. Of these, 1521 were excluded at the title/abstract review stage, leaving 212 papers for full-text review. Of these 212 papers, 84 were excluded as they were not full-text published research articles; 25 did not meet the required sample criteria of at least 75% of the sample being boys or men with DMD; 21 were judged not to be assessing QoL; 16 were not published in English; and finally 11 papers did not feature a multi-item PROM. Five articles were additionally excluded during the review of the actual PROM used in the manuscript for not assessing QoL. Accordingly, a total of 50 records from the initial searches met the selection criteria for Search A. A further 5 articles that met the selection criteria for Search A were added as a result of citation tracking, giving a total of 55 records.

Table 1 summarises the PROMs used to assess QoL in DMD from the full-texts meeting the selection criteria at Stage 1 (*n* = 55). A total of 40 PROMs used to assess at least one aspect of QoL in DMD were identified in published research articles through database searching (the two HUI classification systems use the same 15-item PROM). The majority of the PROMs were multidimensional (*n* = 32), designed to assess a range of different facets of QoL. The remaining unidimensional scales were designed to assess: activity limitations (CALI); anxiety (GAD-7); depression (BDI, DIKJ, PHQ-9); fatigue severity (FSS); life satisfaction (SWLS); or quality of life/health-related quality of life unidimensionally (KIDSCREEN, SOLE). Twenty-four of the PROMs had versions designed for completion by adult or young adult respondents, and 26 had versions designed for children. The most popular PROMs used in published research articles assessing QoL in people with DMD were the PedsQL 4.0 GCS (18 articles); PedsQL 3.0 NMM (10 articles); and the SF-36 (8 articles).

**Table 1:** Patient reported outcome measures assessing quality of life identified in published articles with samples of people with DMD

PROM	Respondent type	Recall Period	***N*** dimensions (items)	Dimensions of quality of life assessed	Response options	Total score range	Origin	Validated English copy and development papers freely available for review?
36-item Short-Form Health Survey (SF-36) v1.0 [30–37]	Adult self-report	Varies by dimension	8 + a single item of perceived change in health (36 items)	Physical functioning; bodily pain; role limitations due to physical health problems; role limitations due to personal or emotional problems; emotional well-being; social functioning; energy/fatigue; general health perceptions	Varies by dimension	No total score calculated	USA	Yes
SF-36 Health Survey v2.0 [38]	Adult self-report	Varies by dimension	8 + a single item of perceived change in health (36 items)	Assumed same as SF-36 v1.0	Varies by dimension	No total score calculated	USA	No
Autoquestionnaire Qualité de vie Enfant Imagé (AUQEI) [39]	Child self-report	Unknown/undefined	4 + a total score (26 items)	Autonomy; leisure; functioning; family; total	0–3 rating scale (with pictures)	0–78 (raw)	France	No
Beck Depression Inventory I (BDI) [34]	Adult interview or self-report	Present/today	1 total score (21 items)	Depression	0–3 rating scale	0–63 (raw)	USA	Yes
Behavior Assessment System for Children (BASC) first edition [40]	Child self-report Parent report Teacher report	Unknown (withdrawn from use, superseded by BASC-II and BASC-III)	Unknown (withdrawn from use, superseded by BASC-II and BASC-III)	Unknown (withdrawn from use, superseded by BASC-II and BASC-III)	Unknown (withdrawn from use, superseded by BASC-II and BASC-III)	Unknown (withdrawn from use, superseded by BASC-II and BASC-III)	USA	No
Child Activity Limitations Interview (CALI) [40]	Child interview or self-report	Last 4 weeks	1 total score (8 items chosen from a set of 21)	Activity limitations	0–4 rating scale	0–32 (raw)	USA	Yes
Child Health Questionnaire - Parent Form 50 (CHQ-PF50) [41, 42]	Parent self-report	Last 4 weeks (past year for change in health)	14 (50 items)	Physical functioning; role/social limitations – physical; role/social limitations – emotional; role/social limitations – behavioral; general health perceptions; bodily pain/discomfort; family activities; parent impact – time; parent impact – emotion; self-esteem; mental health; behaviour; family cohesion; change in health	Varies by dimension	No total score calculated	USA	No
Children’s Assessment of Participation and Enjoyment (CAPE) [43–45]	Child and young adults self-report or interview	Unknown/undefined	5 + a total score (55 items)	Diversity of activities; intensity of activities (frequency of participation); enjoyment of activities; with whom; where; total participation	Varies by dimension	0–55 (raw)	USA	No
Depressions-Inventar für Kinder und Jugendliche (DIKJ) 2nd edition [34]	Child self-report	Unknown/undefined	1 total score (26 items)	Depression	Unknown/undefined	0–46 (raw)	Germany	No
DISABKIDS generic module (DCGM-37) [34]	Child self-report Proxy report	Past 4 weeks	6 + a total score (37 items)	Independence; emotion; social inclusion; social exclusion; limitation; treatment; total	1–4 rating scale	37–148 (raw)	Multi-country	Yes
DISABKIDS – Smileys [34]	Child self-report Proxy report	Assumed same as DCGM-37	Assumed same as DCGM-37 (12 items)	Assumed same as DCGM-37	Assumed same as DCGM-37 (with pictures)	12–48 (raw)	Multi-country	No
Dutch Children AZL/TNO Questionnaire Quality of Life Short Form (DUC-25) [45]	Child self-report	Unknown/undefined	4 + a total score (25 items)	Physical; emotional; social; home functioning; total	0–4 rating scale	0–100 (raw)	Netherlands	No
EuroQoL 5-domain 3-Level (EQ-5D-3L) [46, 47]	Adult self-report Proxy report	Present/today	5 (5 items) + self-rated health VAS	Mobility; self-care; usual activities; pain/discomfort; anxiety/depression	1–3 rating scale	−0.594 – 1 (utility scores)	Multi-country	Yes
Fatigue Severity Scale (FSS) [36]	Adult self-report	Within last week	1 total score (9 items) + global fatigue VAS	Fatigue severity	1–7 rating scale	1–63 (raw)	USA	Yes
Generalized Anxiety Disorder Scale 7-item (GAD-7) [48]	Adult self-report	2 weeks	1 total score (7 items)	Anxiety	0–3 rating scale	0–21 (raw)	USA	Yes
Health Utilities Index Questionnaire mark 2 (HUI-2) 15Q [49, 50]	Child self-report Adult self-report Proxy report	During past 4 weeks	7 (15 items)	Sensation; mobility; emotion; cognition; self-care; pain; fertility	Varies by dimension	−0.03 – 1 (utility scores)	Canada	Yes
Health Utilities Index Questionnaire mark 3 (HUI-3) 15Q [49, 50]	Child self-report Adult self-report Proxy report	During past 4 weeks	8 (15 items)	Vision; hearing; speech; ambulation; dexterity; emotion; cognition; pain	Varies by dimension	−0.36 – 1 (utility scores)	Canada	Yes
Hospital Anxiety and Depression Scale (HADS) [36, 47]	Adult self-report	Last week	2 (14 items)	Anxiety; depression	0–3 rating scale	No total score calculated	UK	Yes
Individualized Neuromuscular Quality of Life Questionnaire (INQOL) [38]	Adult self-report	At the moment	10 + a total score (45 items)	Weakness; locking; pain; fatigue; activities; independence; social relationships; emotions; body image; treatment; total	7-point rating scale, varies by dimension	Scoring unclear	UK	Yes
KIDSCREEN-10 [51]	Child self-report Proxy report	Last week	1 total score (10 items)	Health-related quality of life	1–5 rating scale	10–50 (raw)	Multi-country	Yes
KIDSCREEN-27 [52]	Child self-report Proxy report	Last week	5 (27 items)	Physical well-being; psychological well-being; autonomy and parent relation; social support and peers; school environment	1–5 rating scale	No total score calculated	Multi-country	Yes
KIDSCREEN-52 [53]	Child self-report Proxy report	Last week	10 (52 items)	Physical well-being; psychological well-being; moods and emotions; self-perception; autonomy; parent relation and home life; financial resources; social support and peers; school environment; social acceptance (bullying)	1–5 rating scale	No total score calculated	Multi-country	Yes
Life Satisfaction Index for Adolescents (LSIA) [54–57]	Child and young adults self-report	At present	5 + a total score (45 items)	General well-being; interpersonal relationships; personal development; personal fulfilment; leisure and recreation; total	1–5 rating scale (plus 0 = N/A)	0–225 (raw)	Canada	Yes
Muscular Dystrophy Child Health Index of Life with Disabilities (MDCHILD) [8]	Child self-report	Past 4 weeks	7 + a total score (47 items)	Activities of daily living & independence; positioning, transferring, & mobility; comfort & endurance; emotions & behaviour; social interaction & school; health; your overall quality of life; total	Varies by dimension	0–100 (transformed)	Canada	Yes
Neurological Disorders Quality of Life Questionnaire (Neuro-QoL) [35]	Adult self-report	Varies by dimension	Up to 16 (up to 564 items in item banks)	Ability to participate in social roles and activities; anxiety; bowel function; cognitive function; communication; depression; emotional and behavioral dyscontrol; fatigue; lower extremity function – mobility; positive affect and well-being; satisfaction with social roles and activities; sleep disturbance; sexual function; stigma; upper extremity function – fine motor, ADL; urinary/bladder function	1–5 rating scale	No total score calculated	USA	Yes
Offer Self-Image Questionnaire for Adolescents (OSIQ) [54, 57]	Child and young adult self-report or interview	Unknown/undefined	11 + a total score (130 items)	Impulse control; emotional tone; body and self-image; social relationships; morals; vocational and educational goals; family relationships; mastery of the external world; psychopathology; superior adjustment; total	1–6 rating scale	130–780 (raw)	USA	No
Patient Health Questionnaire 9-item (PHQ-9) [48]	Adult self-report	2 weeks	1 total score (9 items)	Depression	0–3 rating scale	0–27 (raw)	USA	Yes
Pediatric Neurological Disorders Quality of Life Questionnaire (Pediatric Neuro-QoL) [35]	Child self-report	Varies by dimension	Up to 11 (up to 161 items in item banks)	Anger; anxiety; cognitive function; depression; fatigue; lower extremity – mobility; pain; social relations – interaction with adults; social relations – interaction with peers; stigma; upper extremity – fine motor, ADL	1–5 rating scale	No total score calculated	USA	Yes
Pediatric Outcomes Data Collection Instrument (PODCI) [58–61]	Child self-report Proxy report	Varies by dimension	7 (86 items)	Global function & comfort; upper extremity function; physical function and sport; transfers and mobility; comfort; POSNA happy and satisfied; POSNA expectations	Varies by dimension	No total score calculated	USA	Yes
Pediatric Quality of Life Inventory (PedsQL) 3.0 DMD module [62, 63]	Child and young adult self-report Proxy report	Past month or past 7 days (acute version)	4 (18 items)	Daily activities; treatment barriers; worry; communication	0–4 rating scale	No total score calculated	USA	Yes
PedsQL 3.0 Multidimensional fatigue scale (MFS) [63, 64]	Adult self-report Child and young adult self-report Proxy report	Past month or past 7 days (acute version)	3 + a total score (18 items)	General fatigue; sleep/rest fatigue; cognitive fatigue; total fatigue	0–4 rating scale	0–100 (transformed)	USA	Yes
PedsQL 3.0 Neuromuscular module (NMM) [35, 50, 51, 63–69]	Child and young adult self-report Proxy report	Past month or past 7 days (acute version)	3 + a total score (25 items)	About my/my child’s neuromuscular disease; communication; about our family resources; total	0–4 rating scale	0–100 (transformed)	USA	Yes
Pediatric Quality of Life Inventory (PedsQL) 4.0 Generic Core Scales (GCS) [35, 43, 51, 58, 61–65, 67, 70–77]	Adult self-report Child and young adult self-report Proxy report	Past month or past 7 days (acute version)	5 + a total score (23 items)	Physical health; psychosocial health; emotional functioning; social functioning; school functioning; total	0–4 rating scale	0–100 (transformed)	USA	Yes
PedsQL 4.0 Generic Short-form (SF-15) [78]	Adult self-report Child and young adult self-report Proxy report	Past month or past 7 days (acute version)	5 + a total score (15 items)	Physical health; psychosocial health; emotional functioning; social functioning; school functioning; total	0–4 rating scale	0–100 (transformed)	USA	Yes
Pittsburgh Sleep Quality Index (PSQI) [35, 74]	Adult self-report	Past month	8 + a total score (10 items)	Subjective sleep quality; sleep latency; sleep duration; sleep efficiency; sleep disturbance; use of sleep medication; daytime dysfunction; total	Varies by dimension	0–21 (raw)	USA	Yes
Satisfaction with Life Scale (SWLS) [48, 54]	Adult self-report	Undefined/present time	1 total score (5 items)	Life satisfaction	1–7 rating scale	5–35 (raw)	USA	Yes
Strength and Difficulties Questionnaire (SDQ) [79–81]	Child self-report Proxy report	Last 6 months	5 + a total score (25 items) + an impact supplement	Emotional symptoms; conduct problems; hyperactivity/inattention; peer relationship problems; total difficulties; prosocial behaviour	0–2 rating scale	0–40 (raw)	UK	Yes
Strips of Life with Emoticons Questionnaire (SOLE) [82]	Child self-report	Specific scenarios	1 total score (33 items)	Quality of life	0–2 rating scale (with pictures)	0–66 (raw)	Italy	No
TNO-AZL Children’s Quality of Life questionnaire (TACQoL) [83]	Child self-report Proxy report	The last few weeks	7 (56 items)	Physical functioning; motor functioning; independent daily functioning; cognitive functioning and school performance; social contacts; positive moods; negative moods	Varies by dimension	No total score calculated	Netherlands	No
TNO-AZL Adult Quality of Life questionnaire (TAAQoL) [83]	Adult self-report	In the last month	12 (45 items)	Gross motor functioning; fine motor functioning; cognition; sleep; pain; social contacts; daily activities; sex; vitality; happiness; depressive mood; anger	Varies by dimension	No total score calculated	Netherlands	No
World Health Organisation Quality of Life Scale-Brief Version (WHOQOL-BREF) [33, 35–37]	Adult self-report or interview Proxy report	2 weeks	4 (26 items, 24 items make up domain scores)	Physical health; psychological; social relationships; environment	1–5 rating scale	No total score calculated	Multi-country	Yes

References next to PROM names represent published studies where the PROM has been used in a sample of people with DMD. PROM = patient reported outcome measure

### Results of search B – evidence on measurement properties of PROMs

**Fig. 1:**
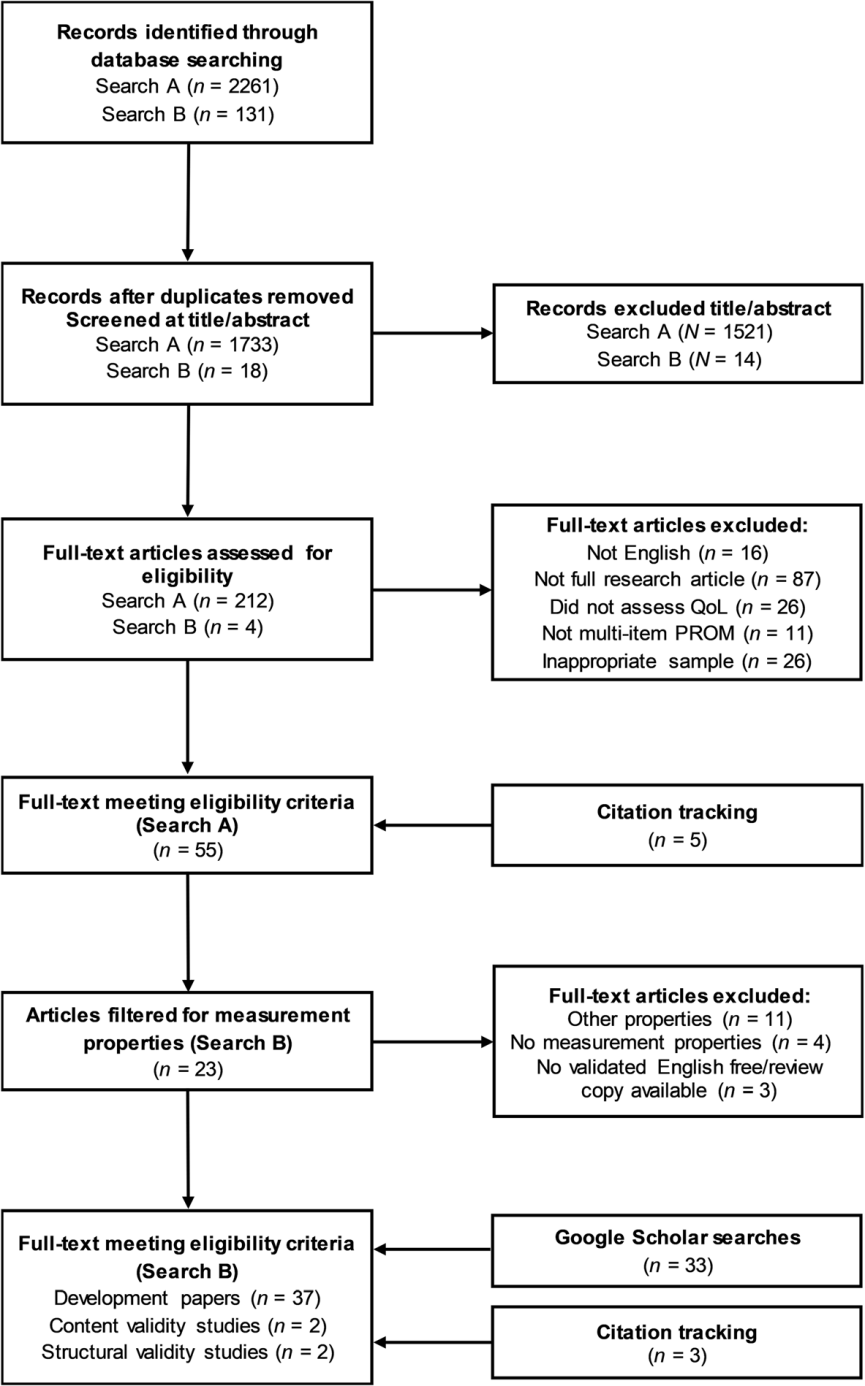
Flow diagram of search strategy and selection of papers

After removing duplicates, 92 records were identified through database searching for Search B. Of these, 51 had already been excluded during Search A. Eighteen unique records were found, 14 were excluded at title/abstract review stage, leaving 4 papers for full-text review. Of these 4 papers, 3 were excluded because they were not full-text primary research articles; and 1 did not meet the sample selection criteria. Of the remaining 23 records that had already met the selection criteria for Search A, 11 were excluded at full-text for containing information on measurement properties other than, and not including, content and structural validity; 4 were excluded as not containing information on measurement properties; and 3 for including a PROM that did not have a validated English copy that was free and/or available for review. The remaining 5 papers that met the selection criteria for Search B featured evidence on content validity (*n* = 3, of which one was classified as a development paper) and structural validity (*n* = 2). Finally, 33 PROM development papers were identified through a review of Google Scholar search results and 3 PROM development papers were identified through citation tracking, resulting in a final selection of 41 papers that met the selection criteria for Search B (see Fig. 1). These included 37 development papers, 2 content validity studies in DMD, and 2 structural validity studies in DMD.

The observed proportionate agreement between reviewers during selection, based on the primary database searches, was 92.4% at title/abstract, with Cohen’s κ = 0.51 or “moderate agreement” and is similar to other published reviews [84, 85]. At full-text review, the observed proportionate agreement was 93.5% with Cohen’s κ = 0.82 or “almost perfect agreement”.

Following the searches, 26 PROMs were taken forward for COSMIN quality assessment on content and structural validity in DMD (Table 2). The remaining 14 PROMs were not assessed for the following reasons: a copy of the PROM itself and/or necessary development papers were not freely accessible for review (CAPE, CHQ-PF50, DISABKIDS Smileys, OSIQ, SF-36 v2); no formally validated English copy of the PROM was available or in use (AUQEI, DIKJ, DUC-25, SOLE, TAAQoL, TACQoL); the PROM was no longer available or recommended for use (BASC 1st edition, which has been superseded by the BASC 2); or it was unclear from the study which of a large number of possible variants of a PROM were used (pediatric Neuro-QoL, Neuro-QoL).

**Table 2:** Characteristics and assessment of development papers for measures included in the review

PROM	Reference(s)	Original language	Construct definition	Target population	Intended context of use	Concept elicitation study	
						COSMIN quality rating	Were patients involved?
BDI	Beck et al. 1961 [86]	English (US)	“the items were chosen on the basis of their relationship to the overt behavioral manifestations of depression and do not reflect any theory regarding the etiology or the underlying psychological processes in depression”	Adult patients with suspected symptoms of depression	Quantitative assessment of the intensity of depression in diagnostic and research settings	Inadequate	No
CALI	Palermo et al. 2004 [87]	English (US)	“functional impairment, defined as difficulty in performing age-appropriate physical, mental, and social activities in daily life due to physical health status (…) functional impairment due to pain (…) specific areas of functioning that are important to children and adolescents with recurrent and chronic pain”	School-age children and adolescents with recurrent and chronic pain	Research and clinical care	Doubtful	Yes
DCGM-37	Petersen et al. 2005 [88] Ravens-Sieberer et al. 2007 [89]	English (UK)	“a multidimensional construct with social, physical, emotional, and functional domains”	Children aged 4–7 years and 8–16 years with chronic health conditions	Clinical studies or surveys	Doubtful	Yes
EQ-5D-3L^a^	EuroQol Group 1990 [6] Brooks et al. 1996 [7]	Multiple, including English (UK)	“Health-related quality of life”	“Large-scale surveys of the community and (…) for use in postal surveys”	“Complement other quality of life measures, collection of common data set for reference. Generate cross-national comparisons of health state valuations.”	Inadequate	No
FSS	Krupp et al. 1989 [90]	English (US)	“Fatigue”	Patients with “clinical disorders”	Clinical research studies and surveys	Inadequate	No
GAD-7	Spitzer et al. 2006 [91]	English (US)	“We first selected potential items for a brief GAD [Generalized Anxiety Disorder] scale (…) that reflected all of the *Diagnostic and Statistical Manual of Mental Disorders, Fourth Edition (DSM-IV)* symptom criteria for GAD and (…) on the basis of review of existing anxiety scales.”	General adult population	Clinical practice and research	Inadequate	No
HADS	Zigmond & Snaith 1983 [92]	English (UK)	“depression subscale were largely based on the anhedonic state (…) psychic manifestations of anxiety neurosis”	Patients under investigation and treatment in medical and surgical departments in non-psychiatric hospital departments	Clinical/screening use within non-psychiatric hospital departments	Inadequate	No
HUI-2 / HUI-3 (15Q)	Feeny et al. 1995 [93] Torrance et al. 1996 [94]	English (US)	“The HUI Mark II and Mark III systems are based on concepts of functional capacity rather than performance (…) generic health profile measures that also permit the computation of a single summary score quantifying health-related quality of life”	Originally survivors of childhood cancer (HUI-2), extended to adults	Clinical evaluative and population health survey studies, in clinical trials, and cost-utility analyses	Inadequate	Unknown
INQoL	Vincent et al. 2007 [95]	English (UK)	“The structure of INQoL was based on the ICIDH-2 model of disease incorporating the concepts of Impairment, Activities, and Participation.”	Adults with neuromuscular disorders (16+ years)	Clinical and research use	Inadequate	Yes
KIDSCREEN-52	Ravens-Sieberer et al. 2001 [96] Ravens-Sieberer et al. 2005 [97] Detmar et al. 2006 [98]	Multiple, including English (UK)	“Health-related quality of life is described as a multidimensional construct covering physical, emotional, mental, social, and behavioral components of well-being and function as perceived by patients and/or individuals (…) agreement was reached that the questionnaire should aim to measure HRQOL as a generic construct in largely healthy children, thus more emphasis was given to the inclusion of psychosocial domains, and less to domains of physical functioning or symptoms such as pain.”	Healthy and chronically-ill children and adolescents between 8 and 18 years	Epidemiological and paediatric studies, clinical settings (healthcare system), and health services research	Adequate	Yes
KIDSCREEN-27	Ravens-Sieberer et al. 2006 [99]	Assumed the same as KIDSCREEN-52	Assumed the same as KIDSCREEN-52	Assumed the same as KIDSCREEN-52	Assumed the same as KIDSCREEN-52	Adequate	Yes
KIDSCREEN-10	Ravens-Sieberer et al. 2006 [99]	Assumed the same as KIDSCREEN-52	Assumed the same as KIDSCREEN-52	Assumed the same as KIDSCREEN-52	Assumed the same as KIDSCREEN-52	Adequate	Yes
LSIA	Reid & Renwick 1994 [54]	English (US)	“quality of life is to conceptualize it as a subjective phenomenon. Specifically, it is viewed in terms of the individual’s feelings and evaluations of his or her life circumstances. Many researchers who study quality of life within this perspective emphasize the importance of measuring the individual’s degree of life satisfaction. In other words, they are interested in how pleased an individual feels about particular aspects of his or her life”	“Individuals between the ages of 12 and 19 years who have DMD”	Research instrument and potentially useful as a clinical measure	Doubtful	Yes
MDCHILD	Propp, 2017 [100] Propp et al. 2019 [8]	English (UK)	“Health-related priorities for children with DMD (…) defined as concerns, desires, and expectations arising from the lived experience of that condition”	Children with DMD (assumed 5–18 years)	Cohort studies, clinical trials, and clinical decision-making	Doubtful	Yes
PedsQL 3.0 DMD	Uzark et al. 2012 [62]	English (US)	“Health-related quality of life (QoL), a multidimensional construct that includes physical, psychological, and social functioning, has emerged as an important outcome in pediatric populations with chronic health conditions.”	Children with DMD from 2 to 18 years	Assumed the same as PedsQL 4.0 GCS	Doubtful	Yes
PedsQL 3.0 MFS	Varni et al. 2002 [101]	English (US)	“designed to measure child and parent perceptions of fatigue in pediatric patients”	Assumed the same as PedsQL 4.0 GCS	“may be utilized as outcome measures in pediatric cancer clinical trials, research, and clinical practice for HRQOL”	Inadequate	Yes
PedsQL 3.0 NMM	Iannaccone et al. 2009 [102]	English (US)	“HRQOL is a multidimensional construct, consisting at the minimum of physical, psychological (including emotional and cognitive), and social health dimensions delineated by the World Health Organization. HRQOL has emerged as the most appropriate term for quality of life dimensions that represent a patient’s perceptions of the impact of an illness and its treatment on their own functioning and well-being and which are within the scope of healthcare services and medical products.”	Children and young people with neuromuscular disorders, in particular spinal muscular atrophy	Assumed the same as PedsQL 4.0 GCS	Doubtful	Yes
PedsQL 4.0 GCS	Varni et al. 1999 [103]	English (US)	“The PedsQL measures the patient’s and the parent’s perceptions of the patient’s HRQOL, as defined in terms of the impact of disease and treatment on an individual’s physical, psychological, and social functioning, and by disease/treatment-specific symptoms.”	Children aged 8–18 across various pediatric chronic health conditions	Epidemiological studies, clinical trials, and performance improvement studies	Doubtful	Yes
PedsQL 4.0 SF-15	Varni et al. 1999 [103]	English (US)	Assumed the same as PedsQL 4.0 GCS	Assumed the same as PedsQL 4.0 GCS	Assumed the same as PedsQL 4.0 GCS	Doubtful	Yes
PHQ-9	Spitzer et al. 1999 [104] Kroenke et al. 2001 [105]	English (US)	“Depression (…) using diagnostic criteria from the *Diagnostic and Statistical Manual of Mental Disorders, Revised Third Edition (DSM-III-R)* and *Diagnostic and Statistical Manual of Mental Disorders, Fourth Edition (DSM-IV).”*	General adult population	Clinical practice and research	Inadequate	No
PODCI	Daltroy et al. 1998 [106]	English (US)	“The POSNA outcomes instrument scales assess upper extremity function, transfers and mobility, physical function and sports, comfort (painfree), happiness and satisfaction, and expectations for treatment. A POSNA global scale combines the three function subscales and comfort.”	Children aged 2–18 years with musculoskeletal disorders	“Patient-based instrument”	Doubtful	Yes (assumed)
PSQI	Buysse et al. 1989 [107]	English (US)	“sleep quality is a readily accepted clinical construct, it represents a complex phenomenon that is difficult to define and measure objectively. ‘Sleep quality’ includes quantitative aspects of sleep, such as sleep duration, sleep latency, or number of arousals, as well as more purely subjective aspects, such as “depth” or “restfulness” of sleep”	Clinical/psychiatric populations	Psychiatric clinical practice and research activities	Doubtful	No
SDQ	Goodman 1997 [108]	English (UK)	“young people’s behaviours, emotions, and relationships”	Children and young people (aged 4–16 years)	“to meet the needs of researchers, clinicians, and educationalists”	Inadequate	No
SF-36 v1.0^a^	Ware & Sherbourne 1992 [109] Hays et al. 1993 [110] Jenkinson et al. 1999 [111] Ware 2000 [112]	English (US)	““Health”, eight concepts: physical functioning, social and role functioning, mental health, general health perceptions, bodily pain, and vitality.”	“General population and patients”	“Clinical practice and research, healthy policy evaluations, and general population surveys”	Inadequate	No
SWLS	Diener et al. 1985 [113]	English (US)	“Life satisfaction refers to a cognitive, judgmental process. Shin and Johnson (1978) define life satisfaction as “a global assessment of a person’s quality of life according to his chosen criteria” (p. 478)”	Unclear	Unclear	Inadequate	No
WHOQOL-BREF	WHOQOL Group 1994 [114] WHOQOL Group 1995 [115] Skevington et al. 1997 [116] WHOQOL Group 1998 [117]	Multiple, including English (UK)	“It is a broad ranging concept incorporating, in a complex way, the person’s physical health, psychological state, level of independence, social relationships, personal beliefs, and relationship to salient features of the environment (…) At minimum, quality of life includes the following dimensions: physical (individuals’ perception of their physical state), psychological (individuals’ perception of their cognitive and affective state) and social (individuals’ perception of the interpersonal relationship relationships and social roles in their life). (…) The WHOQOL includes a spiritual dimension (the person’s perception of ‘meaning in life’, or the overarching personal beliefs that structure and qualify experience).”	“assess the quality of life of chronic disease sufferers, informal caregivers of the sick and disabled, people living in high-stress conditions like refugees, and ‘healthy’ people”	“in routine clinical work, large scale epidemiological studies and in clinical trials”	Doubtful	Yes

^a^PROM development information from prior COSMIN review [118], not re-extracted or re-rated in this review, based on COSMIN guidance [13]. PROM = patient reported outcome measure; COSMIN = COnsensus-based Standards for the selection of health Measurement INstruments

### Content validity – appraisal of PROM development studies

Table 2 summarises key characteristics and COSMIN quality assessment of the development of the PROMs included in the review. Five PROMs were developed to be intended for use specifically within neuromuscular disorders (INQoL, PedsQL 3.0 NMM) or DMD (LSIA, MDCHILD, PedsQL 3.0 DMD module). Eleven PROMs either had no patients involved in their development, or it was unclear if patients were involved.

The joint most common COSMIN quality rating assigned to the PROMs for concept elicitation was inadequate (*n* = 12). This was primarily due to: the PROM development study not being performed in a sample of patients representing the target population (BDI, EQ-5D-3L, GAD-7, HADS, HUI 15Q, PedsQL 3.0 MFS, PHQ-9, SDQ, SF-36, and SWLS); or inadequacies within the details of the qualitative methods used (FSS, INQoL). The concept elicitation study of 11 further PROMs was rated as doubtful due to at least some unclear details/suspected problems within the qualitative methods used (CALI, DCGM-37, LSIA, MDCHILD, PODCI, PedsQL 3.0 NMM, PedsQL 3.0 DMD, PedsQL 4.0 GCS, PedsQL 4.0 SF-15, PSQI, WHOQOL-BREF). Only the KIDSCREEN family of measures (*n* = 3) received an adequate rating for concept elicitation and PROM design. However, the KIDSCREEN measures received a doubtful rating for the overall PROM development study, for failing to provide evidence that comprehensibility and comprehensiveness were assessed in the cognitive interview/pilot study of the PROM.

### Content validity – appraisal of content validity studies

Only 2 published articles had independently assessed the content validity of the QoL PROMs in samples of people with DMD (Table 3). Neither of these studies were conducted in an English language context, and instead were cross-cultural validation studies. Hu et al. (2013) [67] assessed the relevance, comprehensiveness, and comprehensibility of the PedsQL 3.0 NMM in Chinese children with DMD. Simon et al. (2017) [56] assessed comprehensibility of the LSIA in Brazilian children with DMD, and comprehensiveness in professionals. However, both of these studies received ratings of doubtful due to at least some unclear details/suspected problems within the qualitative methods used.

**Table 3:** Characteristics and assessment of content validity papers in DMD samples for measures included in the review

PROM	Reference	Language (Country)	DMD sample characteristics			COSMIN rating			Results (summary)
			N	Age (years, ±SD)	% ambulatory	Relevance	Comprehensiveness	Comprehensibility	
LSIA	Simon et al. 2017 [56]	Brazilian Portuguese (Brazil)	43	11.4 **±** 3.38	Not stated	/	Doubtful	Doubtful	The level of comprehension reached via the final Probe technique was 97% for the parent version and 95% for the patient version, which is above the minimum of 85% required.
PedsQL 3.0 NMM	Hu et al. 2013 [67]	Chinese (China)	56	7.54 **±** 4.06	37 children “could climb stairs”	Doubtful	Doubtful	Doubtful	Cognitive debriefing was conducted with six children with DMD and their parents to confirm that the final Chinese version was understandable and acceptable.

/ = content validity aspect not evaluated. PROM = patient reported outcome measure; COSMIN = COnsensus-based Standards for the selection of health Measurement INstruments

### Content validity evidence synthesis

The evidence from the PROM development papers and content validity studies was combined with reviewer ratings of the PROMs to produce a synthesis of the available evidence using the 10 COSMIN criteria for good content validity [13]. Most of the quality of the evidence was downgraded from High to Low or Very Low due to the assessment being based on development studies of doubtful or inadequate quality, respectively [13]. Only the LSIA and the PedsQL 3.0 NMM had moderate supporting evidence, featuring independent content validity studies as well as development papers. The KIDSCREEN measures and the LSIA were the only PROMs to receive satisfactory results for all three dimensions of content validity: relevance; comprehensiveness; and comprehensibility, based on the evidence available. Full synthesised results are presented in Table 4.

**Table 4:** Evidence synthesis on the content and structural validity of measures that have been used to assess quality of life in people with DMD

	Content validity				Structural Validity	
PROM	Relevance	Comprehensiveness	Comprehensibility	Quality of evidence	Rating of results	Quality of evidence
BDI	±	–	±	Very low	?	?
CALI	+	–	±	Low	?	?
DCGM-37	±	+	+	Low	?	?
EQ-5D-3L	+	–	+	Very low	?	?
FSS	±	–	±	Very low	?	?
GAD-7	+	–	+	Very low	?	?
HADS	–	–	–	Very low	?	?
HUI-2 / HUI-3 (15Q)	–	–	–	Very low	?	?
INQoL	±	±	+	Very low	?	?
KIDSCREEN-52	+	+	+	Low	?	?
KIDSCREEN-27	+	+	+	Low	?	?
KIDSCREEN-10	+	+	+	Low	?	?
LSIA	+	+	+	Moderate	?	?
MDCHILD	±	+	+	Low	?	?
PedsQL 3.0 DMD	±	?	±	Very low	?	?
PedsQL 3.0 MFS	±	–	±	Very low	?	?
PedsQL 3.0 NMM	±	?	±	Moderate	–	High
PedsQL 4.0 GCS	±	+	±	Low	?	Very low
PedsQL 4.0 SF-15	±	+	±	Low	?	?
PHQ-9	+	–	±	Very low	?	?
PODCI	±	+	±	Very low	?	?
PSQI	±	–	±	Very low	?	?
SDQ	–	–	+	Very low	?	?
SF-36 v1.0	±	+	±	Very low	?	?
SWLS	–	–	±	Very low	?	?
WHOQOL-BREF	+	+	±	Very low	?	?

+ = satisfactory results; − = unsatisfactory results; ± = inconsistent results;? = indeterminate. PROM = patient reported outcome measure

### Structural validity - appraisal of structural validity studies

Two studies had assessed the structural validity of the PROMs included in this review in samples of people with DMD (Table 5). Both of these were conducted using English versions of the PROMs and either in the UK or USA. Lim et al. (2014) [72] assessed the structural validity of the PedsQL 4.0 GCS using an unspecified Rasch model in 63 boys with DMD. This study received a COSMIN quality rating of doubtful because it was doubtful that the sample size included in the analysis was adequate. Landfeldt et al. (2018) [66] assessed the structural validity of the PedsQL 3.0 NMM using a Rasch partial-credit model (PCM) in 278 people with DMD. This study received a very good COSMIN quality rating for its methodological content.

**Table 5:** Characteristics, assessment, and results of structural validity papers in DMD samples for measures included in the review

PROM	Reference	Country (language)	Patient characteristics				COSMIN Quality Rating	Analysis – model	Results (summary)
			N	Age (yr, M ± SD)	% ambulatory	PROM score			
PedsQL 4.0 GCS	Lim et al. 2014 [72]	USA (English)	63 boys with DMD (and up to 50 parents, not necessarily matched)	10.2 ± 2.5	95.24	Child: M = 64.5, SD = 15.3. Parent: M = 56.2, SD = 12.9.	Doubtful	Rasch (model not specified)	Model misfit for items determined with infit > 1.4 and outfit > 2.0 MnSq values and standardized scores > 2.0. All items fit in parent proxy-reports of physical health scale and child self-reports of psychosocial health scale. 2 out of 8 items showed high infit statistics in child self-reports of the physical health scale (taking a bath or shower; doing chores around the house). In addition 2 out of 15 items showed high infit for the parent proxy-reports of the psychosocial health scale (trouble sleeping; keep up with school work).
PedsQL 3.0 NMM	Landfeldt et al. 2018 [66]	UK / USA (English)	278 (95 UK)	16 ± 7	40% not “full-time wheelchair dependent”	Not reported	Very good	Rasch PCM	Eight items displayed inadequate fit (χ^2^: *p* > 0.01). Six items had fit residuals ≤ −2.5 or ≥ 2.5 (4 significant at *p* < .05). Inadequate overall fit (χ^2^ item-trait interaction: *p* = < .001). Disordered thresholds for 22 of 25 items. Suboptimal targeting.

PROM = patient reported outcome measure; COSMIN = COnsensus-based Standards for the selection of health Measurement INstruments

### Structural validity evidence synthesis

Of the 2 studies that assessed the structural validity of the PedsQL 4.0 GCS and PedsQL 3.0 NMM in people with DMD, neither provided satisfactory results (Table 4). First, the structural validity of the PedsQL 4.0 GCS in people with DMD received an indeterminate rating, as key details of the results from the Rasch model denoting good measurement properties were not reported. Due to the risk of bias assessment of Lim et al. (2014) [72] the quality of the evidence supporting this indeterminate conclusion was rated as very low. Second, the structural validity of the PedsQL 3.0 NMM in people with DMD received an unsatisfactory rating, as the psychometric criteria for good measurement properties were not met. The favourable risk of bias assessment for Landfeldt et al. (2018) [66] meant that the quality of evidence supporting this conclusion was graded as high.

### Quality assurance of the review

The quality of this review was self-assessed against a newly derived COSMIN checklist [22], designed to evaluate the quality of systematic reviews of health-related PROMs. The results are displayed in Additional file 3.

In general, the review meets numerous quality indicators as defined by the COSMIN team, including the elements included in the research aim, search strategies, article selection, and assessment of measurement properties and quality. In a couple of instances, criteria have been partly met. For example, in this review all instruments were included where a *validated English copy was freely available for review*. It is possible that additional instruments could have been included if licenses were paid for to access the relevant PROMs and development materials, and this could be considered a limitation. Second, citation tracking (i.e. reference checking) was conducted on the final set of articles eligible at Stage 2 of the searches (*n* = 41), but not on results eligible for inclusion at Stage 1.

## Discussion

In this systematic review, the published scientific evidence on the content and structural validity of PROMs used to measure at least one aspect of QoL in people with DMD was thoroughly evaluated. The overriding theme was one of sparse evidence. Many PROMs that are being used to assess aspects of QoL in people with DMD are being utilised without the accompanying good quality evidence that supports their validity for this task. Only five of the PROMs uncovered in this review were specifically designed for use in people with neuromuscular problems (three for DMD), and only two of these have had their content and/or structural validity independently assessed in this population (with the content validity studies involving translated versions). When the evidence is available, most of it is either of a low quality, featuring insufficient detail in the published articles to make thorough and comprehensive assessments of content and structural validity as demanded by COSMIN [16], leading to doubtful ratings. Indeed, one of the highest quality pieces of evidence reviewed in terms of reported methodology, Landfeldt et al. (2018) [66], reported insufficient structural validity of the PedsQL 3.0 Neuromuscular module (NMM) in DMD.

The results from the review may not be viewed as surprising. Many of the PROMs identified are what could be described as “legacy” measures. They were developed at a time when the science of construct and item generation was largely overlooked. The content of instruments was largely defined by clinical or expert opinion, with little explanation of what that entailed. The reporting of such stages in publications or questionnaire manuals was not commonplace. The transparency of reporting on the early stages of PROM development has only gained traction in the last decade or so. Whilst this is a positive step for researchers, clinicians and users alike, progress can be limited by journal restrictions on word count and remit. It is however possible for such legacy measures to be appropriately validated (or have their validity assessed) in properly designed studies assessing content or structural validity in modern samples of people with DMD. The problem observed in this review is that researchers are likely using such measures as a consequence of precedent or tradition, rather than a supportive evidence base.

Another related legacy issue within PROM development, which this review touches upon and has changed for the better over time, is a recognition of the importance of direct patient involvement in developing PROMs [119, 120]. In this review, almost half (11 out of 26) of the PROMs did not demonstrate any evidence of patient involvement in their development. While most of these PROMs are legacy measures, this is a noteworthy figure, given that patient involvement is the only way to ensure a PROM is capturing health and QoL outcomes in a way that is relevant, comprehensive, and comprehensible to the patient population [119]. The use of patient involvement in PROM development is thus advantageous for researchers and patients alike. To help guide PROM developers, a recent framework has been published to help researchers fully incorporate patient and public involvement (PPI) in the development of PROMs moving forward [120].

In the current review, some PROMs performed better than others under COSMIN assessment. First, the KIDSCREEN instrument (all versions) does show some evidence of applicability given that it covers many aspects of QoL. The PROM development study for the KIDSCREEN instrument was the only one rated as adequate, it was designed to assess QoL in children and adolescents with chronic illnesses, and the ratings for the content validity of the measure were positive (based on the available evidence in the measure’s development). However, it must also be borne in mind that there is little or no direct evidence to support the content or structural validity in DMD, specifically. The original KIDSCREEN instrument (52-item version) was designed to assess multiple aspects of QoL, namely: physical well-being; psychological well-being; moods and emotions; self-perception; autonomy; parent relation and home life; financial resources; social support and peers; school environment; and social acceptance (bullying), covering much of the CMQM framework [9]. The conceptual framework of the instrument is thus intuitively applicable to the Duchenne community; however the measurement of impact may be limited due to the target age range of the PROM itself (8–18 years). While this is not uncommon (i.e. differences in measuring QoL from child to adulthood), there is some question of the applicability for the broader DMD population given the lower age target.

The second-best performing PROM in this review was the LSIA, which received a satisfactory score for relevance, comprehensiveness, and comprehensibility in terms of content validity, based on the information available and reviewers’ ratings of the PROM itself. However, the development study for this paper lacked key details necessary in good PROM development, and thus was rated as doubtful. Furthermore, while the LSIA was one of few measures to feature a content validity study, it was a cross-cultural adaptation study of a Brazilian version of the measure, and the results of the formal assessment of this study were doubtful. While the measure is comprehensive, it only comes in a 45-item version, which is potentially quite burdensome. Furthermore, the measure is designed for use in children and young adults only, and may not generalise to adults with DMD.

The most recent PROM developed specifically for use in children and adolescents with DMD was the MDCHILD. Although the PROM is designed to measure “health-related priorities” [8], much of the content maps onto the CMQM framework [9] and thus covers QoL. While the MDCHILD had many commendable strengths in PROM design, the overall rating of the PROM development, based on the COSMIN worst score counts system [25], was rated as doubtful due to lack of details reported in the development papers. For example, it was unclear if skilled interviewer(s) were used; to what degree data was coded independently; and to what degree, if at all, at least two researchers were involved in the data analysis. This led to a low quality of evidence. Further, because the target population of interest was not clearly defined (i.e. age ranges were not specified), despite performing well in other areas, the PROM received an inconsistent rating for relevance. These results speak to the potential harshness of a worst score counts system advocated by COSMIN, which we discuss further below. Further, because the PROM is new, there is a lack of published content validity studies that may improve the quality of evidence for the MDCHILD going forward, such as that contained in a non-peer-reviewed thesis [100], not eligible for inclusion in the current review.

The PedsQL and associated modules were the most commonly used out of all the PROMs identified within the review. It should be noted that the development studies of the PedsQL were rated as doubtful. There was little evidence to support the content validity of the neuromuscular module of the PedsQL 3.0 (NMM). Furthermore, the psychometric properties of the NMM were not well supported by Landfeldt et al. (2018) [66]. The inclusion of PedsQL within clinical practice, cohort studies or pragmatic trials in DMD thus appears to be based upon precedent and common use, rather than published empirical evidence of suitability, based on content and structural validity. A notable advantage of the PedsQL (and its derivatives) is the young child (via proxy report), child (self and proxy report), young adult forms (self-report), and adult forms, which have now been developed. A further consideration is that the PedsQL scales are designed to be used in parallel (e.g. the generic core scales with the NMM or DMD modules), but were assessed individually under COSMIN guidance. Thus comprehensiveness may be improved by using these scales together.

The search identified some PROM instruments that we were unable to obtain. Access to the PROM and/or associated development papers was limited due to licensing requirements, and therefore it was not possible to include these instruments within the review. It is unlikely that these instruments are commonly used within research and/or clinical practice due to the difficulties around access. Their suitability for the DMD population cannot formally be determined; however, their use is likely to be limited by a lack of accessibility derived from license restrictions, reflected in the few citations in which they appeared.

This review adopted guidance developed by the COSMIN initiative, and has adhered to their recommended methods in identification of evidence, data extraction, data assessment and data synthesis. Whilst the appropriateness of these robust methods cannot be questioned, this has resulted in relatively low ratings of the PROMs included within the review. It is important to recognise that this does not suggest categorically that the instruments used within published and/or current studies are not appropriate or fit for purpose; content and structural validity only form one component of PROM suitability within a population. Furthermore, as stated, many of the instruments were developed at a time when instrument development methods and procedures were not reported – that is not to say the development of the instruments is flawed, just that an assessment of them cannot be made. The COSMIN appraisal tools assume a worst score counts system for the rating of the methodological quality of studies [25]. This means that, in theory, a study could be rated as very good or adequate on all but one criteria, on which it is rated as doubtful or inadequate, and the overall score is thus reduced to the latter lower-quality rating. Sometimes this can be because key details, such as whether skilled interviewers were used, are not reported.

This review is not without its limitations. While the methodological approach of the review is robust and follows the recommendations of COSMIN and that of other published reviews, it must be acknowledged that the rating criteria of the PROMs identified can be viewed as harsh. The COSMIN approach encourages researchers and reviewers to critically appraise evidence of PROM development – however the presence of evidence within published literature is sparse. That is not to say that the development phases did not occur, merely that they are not reported and/or not reported in sufficient detail as required by COSMIN assessment. To critique a PROM’s applicability using this criterion could be perceived as being unduly critical; more recent PROMs tend to report the early stages of instrument development, and we are assessing all PROMs by modern standards. Similarly, the descriptions of PROMs themselves are often lacking. Basic information such as number of items, recall period, domain structure and scoring procedure were noted to be sporadically reported, although better in recent literature. The COSMIN-recommended reviewer rating of the identified PROMs for suitability for DMD (as reported in Table 4) has a large subjective component. Whilst this was completed as per the COSMIN guidelines (with two reviewers and discrepancies reconciled following discussion), some of the ratings are at risk of bias based on the team of raters (i.e. QoL researchers). For example, it is not known whether similar ratings of suitability would be achieved if reviewed by an individual with DMD, a family member or carer of a person with DMD, or a clinician, and we recommend that PPI is incorporated in future COSMIN reviews of content validity. This is further exacerbated when we consider what QoL is – for the purpose of this review it was a multidimensional construct, PROMs that measure a subset of interest (such as depression) may be appropriate to include within studies as part of a host/suite of measures.

The focus of this review was to report on the content and structural validity of PROM instruments that have been used to quantify the impact of DMD on individuals’ QoL. However, content and structural validity only address some aspects of PROM suitability, and further work could be undertaken to formally appraise the instruments described. Other measurement properties, such as psychometric performance, could be considered. Given that DMD is a rare condition, the development and validation of PROMs that measure the impact of the condition on QoL is challenging. The number of participants included within various phases of PROM development and validation will be lower than that of a condition such as diabetes, asthma or eczema. Accordingly, the inclusion of subsidiary samples such as other neuromuscular disorders, may be of interest. However, it is not known how appropriate this would be. It can be postulated that other neuromuscular disorders could imply similar impacts upon QoL, however this has not been explored within the context of this review.

## Conclusions

In conclusion, evidence on the content and structural validity of PROMs assessing QoL in DMD is lacking. Accordingly, our first recommendation from this review is for more research into the content and structural validity of QoL PROMs used in DMD, and, if PROMs are found to be insufficient on these criteria, for additional PROM development within DMD. Second, as the result of this COSMIN assessment, without further direct content validation work in DMD, we would provisionally recommend the KIDSCREEN for measuring QoL in children and adolescents with DMD. Nonetheless, we caution that the KIDSCREEN has not been formally validated in samples of people with DMD. Accordingly, more research is needed to definitively support the continued use of KIDSCREEN (and its derivatives) within DMD. Finally, in the absence of further evidence, it is difficult to recommend the routine use of a measure to assess QoL in adults with DMD on content and structural grounds. Instead, the findings of this review support the need for further PROM development, which is able to accurately assess the impact of DMD on QoL.

## Supplementary information

**Additional file 1.** Full search strategies. Tables showing the full search strategy used in the review.

**Additional file 2.** Example COSMIN ratings KIDSCREEN-52. Example COSMIN content validity rating spreadsheet for the KIDSCREEN-52.

**Additional file 3.** Quality assurance of the review. Quality assessment of the systematic review against COSMIN guidance.

## Data Availability

Data sharing is not applicable to this article as no datasets were generated or analysed during the current study.

## Abbreviations

AUQEI: Autoquestionnaire Qualité de vie Enfant Imagé
BASC: Behavior Assessment System for Children
BDI: Beck Depression Inventory
CALI: Child Activity Limitations Interview
CAPE: Children’s Assessment of Participation and Enjoyment
CFA: Confirmatory factor analysis
CFI: Comparative fit index
CHQ-PF50: Child Health Questionnaire - Parent Form 50
CMQM: Comprehensive Model of QoL in Muscular Dystrophy
COSMIN: COnsensus- based Standards for the selection of health Measurement INstruments
DCGM-37: DISABKIDS generic module
DIKJ: Depressions-Inventar für Kinder und Jugendliche
DMD: Duchenne muscular dystrophy
DUC-25: Dutch Children AZL/TNO Questionnaire Quality of Life Short Form
EQ-5D(− 3 L): Euroqol instrument, Euroqol-5 dimension (3 level)
EQ-5D-Y: Euroqol instrument, Euroqol-5 dimension (youth version)
FSS: Fatigue Severity Scale
GAD-7: Generalized Anxiety Disorder Scale 7-item
GRADE: Grading of Recommendations, Assessment, Development and Evaluations
HADS: Hospital Anxiety and Depression Scale
HUI: Health Utilities Index
INQOL: Individualized Neuromuscular Quality of Life Questionnaire
IRT: Item response theory
LSIA: Life Satisfaction Index for Adolescents
MDCHILD: Muscular Dystrophy Child Health Index of Life with Disabilities
Neuro-QoL: Neurological Disorders Quality of Life Questionnaire
OSIQ: Offer Self-Image Questionnaire
PBM: Preference based measure
PedsQL 3.0 DMD: Pediatric Quality of Life Inventory (PedsQL) 3.0 DMD module
PedsQL 3.0 MFS: PedsQL 3.0 Multidimensional fatigue scale
PedsQL 3.0 NMM: PedsQL 3.0 Neuromuscular module
PedsQL 4.0 GCS: Pediatric Quality of Life Inventory (PedsQL) 4.0 Generic Core Scales
PedsQL 4.0 SF-15: PedsQL 4.0 Generic Short-form
PHQ-9: Patient Health Questionnaire Depression Scale
PODCI: Pediatric Outcomes Data Collection Instrument
PROM: Patient reported outcome measure
PROSPERO: International Prospective Register of Systematic Reviews
PSQI: Pittsburgh Sleep Quality Index
QoL: Quality of life
Rasch PCM: Rasch Partial-credit model
RMSEA: Root mean square error of approximation
SDQ: Strengths and Difficulties Questionnaire
SF-36: 36-item Short-Form Health Survey
SOLE: Strips of Life with Emoticons Questionnaire
SRMR: Standardized root mean squared residual
SWLS: Satisfaction with Life Scale
TAAQoL: TNO-AZL Questionnaire for Adult’s Health-Related Quality of Life
TACQOL: TNO-AZL Questionnaire for Children’s Health-Related Quality of Life
TLI: Tucker Lewis index
WHOQOL-BREF: World Health Organisation Quality of Life Scale-Brief Version

## Footnotes

1 A less-restrictive and simplified version of this filter was applied to the Cochrane Library and CINAHL searches, due to a low number of hits prior to filtering for the measurement properties (see Additional file 1 for full search strategies).
