# Supplementary material for: Measuring quality of life in Duchenne muscular dystrophy: a systematic review of the content and structural validity of commonly used instruments
Source: Health Qual Life Outcomes. 2020 Aug 3;18:263. doi: 10.1186/s12955-020-01511-z (PMC7397669; doi:10.1186/s12955-020-01511-z)
Supplement: Supplementary file 1 — Additional file 1. Full search strategies. Tables showing the full search strategy used in the review. [file 12955_2020_1511_MOESM1_ESM.docx]

**Additional file 1: Full search strategies**

**Table A.1. Database: Ovid MEDLINE Search A. Platform: Ovid SP**

|  | **Query** | **Results** |
| --- | --- | --- |
| #1 | Muscular Dystrophy, Duchenne/ | 4700 |
| #2 | duchenne*.mp. | 11822 |
| #3 | #1 or #2 | 11822 |
| #4 | (HR-PRO or HRPRO or HtaRQL or HRQoL or QL or QoL).ti,ab. or quality of life.mp. or (health index* or health indices or health profile*).ti,ab. or health status.mp. or ((patient or self or child or parent or carer or proxy) adj (appraisal* or appraised or report or reported or reporting or rated or rating* or based or assessed or assessment*)).ti,ab. or ((disability or function or functional or functions or subjective or utility or utilities or wellbeing or well being) adj2 (index or indices or instrument or instruments or measure or measures or questionnaire* or profile or profiles or scale or scales or score or scores or status or survey or surveys)).ti,ab. | 649346 |
| #5 | 'Pediatric Quality of Life Inventory'.mp. ( | 884 |
| #6 | PedsQL.mp. | 1094 |
| #7 | (SF-36 or EQ-5D*).mp. | 24322 |
| #8 | "World Health Organization Quality of Life".mp. | 1373 |
| #9 | (KIDSCREEN or Pittsburgh Sleep Quality questionnaire or PSQI).mp. [mp=title, abstract, original title, name of substance word, subject heading word, floating sub-heading word, keyword heading word, protocol supplementary concept word, rare disease supplementary concept word, unique identifier, synonyms] | 2710 |
| #10 | "Childrens Assessment of Participation and Enjoyment".mp. | 96 |
| #11 | (CAPE or "Child Health Questionnaire" or CHQ* or Health Utilities Index Questionnaire or HUI*).mp.11 | 17397 |
| #12 | (Fatigue Severity Scale or FSS or "Hospital Anxiety and Depression Scale" or HADS or COPE Inventory or "Quality of Life in Neuromuscular Disease").mp. | 10638 |
| #13 | (QoL-NMD DISABKIDS or "Depressionsiventar fur kinder und Jugendliche" or DIKJ or Beck Depression Inventory or BDI or CARE-NMD or State-Trait Anxiety Inventory or STAI or "Life Satisfaction Index for Adolescents" or LSI-A or Quality of Life Evaluation Scale or AUQUEI or Activity Limitations Questionnaire or ACTIVLIM).mp. | 17368 |
| #14 | (sf12 or sf 12 or short form 12 or shortform 12 or sf twelve or sftwelve or shortform twelve or short form twelve).mp. | 4980 |
| #15 | (sf36 or sf 36 or short form 36 or shortform 36 or sf thirtysix or sf thirty six or shortform thirtysix or shortform thirty six or short form thirtysix or short form thirty six).mp. | 23191 |
| #16 | #5 or #6 or #7 or #8 or #9 or #10 or #11 or #12 or #13 or #14 or #15 | 80226 |
| #17 | #4 or #16 | 685940 |
| #18 | #3 and #17 | 523 |

*Note*. The Oxford PROM filter is #4.

**Table A.2. Database: Ovid MEDLINE Search B. Platform: Ovid SP**

|  | **Query** | **Results** |
| --- | --- | --- |
| #1 | Muscular Dystrophy, Duchenne | 4638 |
| #2 | duchenne*.mp. | 11693 |
| #3 | #1 or #2 | 11693 |
| #4 | Autoquestionnaire Qualite de vie Enfant Image.mp. | 18 |
| #5 | (Behavior Assessment System for Children or BASC or Parent Form 50 or PF50 or DUX-25 or EuroQoL 5-domain or Functional Independence Measure* or FIM).mp. | 4877 |
| #6 | (WeeFIM or Life Satisfaction Index or LSI or LSIA or Neurological Disorders Quality of Life Questionnaire or NeuroQOL or pediatric NeuroQOL or Offer Self-Image Questionnaire for Adolescents or OSIQ).mp. | 1602 |
| #7 | (Pediatric Outcomes Data Collection Instrument or PODCI or Neuromuscular module or DMD module or Multidimensional Fatigue Scale or Generic Short-Form or SF15).mp. | 411 |
| #8 | (SDQ or 'Strips of Life with Emoticons Questionnaire' or SOLE or 'World Health Organisation Quality of Life Scale-Brief Version' or WHOQOL-BREF).mp. | 53728 |
| #9 | ('Strength and Difficulties Questionnaire').mp. [mp=title, abstract, original title, name of substance word, subject heading word, floating sub-heading word, keyword heading word, protocol supplementary concept word, rare disease supplementary concept word, unique identifier, synonyms] | 223 |
| #10 | "Individualized Neuromuscular Quality of Life Questionnaire".mp. | 9 |
| #11 | (INQOL or Child Activity Limitations Interview or CALI or "Satisfaction with Life Scale" or SWLS).mp. | 1461 |
| #12 | 'Pediatric Quality of Life Inventory'.mp. | 871 |
| #13 | ("Pediatric Orthopedic Society of North America Pediatric Musculoskeletal Functional Health Questionnaire" or POSNA or Pittsburgh Sleep Quality Index).mp. | 3411 |
| #14 | PedsQL.mp. | 1078 |
| #15 | (SF-36 or EQ-5D*).mp. | 23965 |
| #16 | "World Health Organization Quality of Life".mp. | 1349 |
| #17 | (KIDSCREEN or Pittsburgh Sleep Quality questionnaire or PSQI).mp. [mp=title, abstract, original title, name of substance word, subject heading word, floating sub-heading word, keyword heading word, protocol supplementary concept word, rare disease supplementary concept word, unique identifier, synonyms] | 2640 |
| #18 | "Childrens Assessment of Participation and Enjoyment".mp. | 96 |
| #19 | (CAPE or "Child Health Questionnaire" or CHQ* or Health Utilities Index Questionnaire or HUI*).mp. | 17206 |
| #20 | (Fatigue Severity Scale or FSS or "Hospital Anxiety and Depression Scale" or HADS or COPE Inventory or "Quality of Life in Neuromuscular Disease").mp. | 10441 |
| #21 | (QoL-NMD DISABKIDS or "Depressionsiventar fur kinder und Jugendliche" or DIKJ or Beck Depression Inventory or BDI or CARE-NMD or State-Trait Anxiety Inventory or STAI or "Life Satisfaction Index for Adolescents" or LSI-A or Quality of Life Evaluation Scale or AUQUEI or Activity Limitations Questionnaire or ACTIVLIM).mp. | 17136 |
| #22 | (sf12 or sf 12 or short form 12 or shortform 12 or sf twelve or sftwelve or shortform twelve or short form twelve).mp. | 4886 |
| #23 | (sf36 or sf 36 or short form 36 or shortform 36 or sf thirtysix or sf thirty six or shortform thirtysix or shortform thirty six or short form thirtysix or short form thirty six).mp. | 22934 |
| #24 | #4 or #5 or #6 or #7 or #8 or #9 or #10 or #11 or #12 or #13 or #14 or #15 or #16 or #17 or #18 or #19 or #20 or #21 or #22 or #23 | 140059 |
| #25 | #3 and #24 | 82 |
| #26 | #25 and COSMIN measurement properties filter (see Table A.3) | 36 |

**Table A.3. Database: Ovid MEDLINE Search B Terwee (COSMIN) filter. Platform: Ovid SP**

|  | **Query** |
| --- | --- |
| #1 | instrumentation.sh. |
| #2 | methods.sh. |
| #3 | Validation Studies.pt. |
| #4 | Comparative Study.pt. |
| #5 | Psychometrics/ |
| #6 | psychometr*.ti,ab. |
| #7 | clinimetr*.tw. |
| #8 | clinometr*.tw. |
| #9 | "Outcome Assessment (Health Care)"/ |
| #10 | outcome assessment.ti,ab. |
| #11 | outcome measure*.tw. |
| #12 | Observer Variation/ |
| #13 | observer variation.ti,ab. |
| #14 | Health Status Indicators/ |
| #15 | "Reproducibility of Results"/ |
| #16 | reproducib*.ti,ab. |
| #17 | Discriminant Analysis/ |
| #18 | reliab*.ti,ab. |
| #19 | unreliab*.ti,ab. |
| #20 | valid*.ti,ab. |
| #21 | coefficient.ti,ab. |
| #22 | homogeneity.ti,ab. |
| #23 | homogeneous.ti,ab. |
| #24 | "internal consistency".ti,ab. |
| #25 | 1 or 2 or 3 or 4 or 5 or 6 or 7 or 8 or 9 or 10 or 11 or 12 or 13 or 14 or 15 or 16 or 17 or 18 or 19 or 20 or 21 or 22 or 23 or 24 |
| #26 | cronbach*.ti,ab. |
| #27 | (alpha or alphas).ti,ab. |
| #28 | 26 and 27 |
| #29 | item.ti,ab. |
| #30 | (correlation* or selection* or reduction*).ti,ab. |
| #31 | 29 and 30 |
| #32 | (agreement or precision or imprecision or "precise values" or "test–retest").ti,ab. |
| #33 | (test and retest).ti,ab. |
| #34 | reliab*.ti,ab. |
| #35 | (test or retest).ti,ab. |
| #36 | 34 and 35 |
| #37 | 28 or 31 or 32 or 33 or 36 |
| #38 | (stability or interrater or inter-rater or intrarater or intra-rater or intertester or inter-tester or intratester or intra-tester or interobserver or inter-observer or intraobserver or intra-observer).ti,ab. |
| #39 | (intertechnician or inter-technician or intratechnician or intra-technician or interexaminer or inter-examiner or intraexaminer or intra-examiner or interassay or inter-assay or intraassay or intra-assay or interindividual or inter-individual or intraindividual or intra-individual or interparticipant or inter-participant or intraparticipant or intra-participant).ti,ab. |
| #40 | (kappa or "kappa’s" or kappas or repeatab*).ti,ab. |
| #41 | repeatab*.ti,ab. |
| #42 | 38 or 39 or 40 or 41 |
| #43 | (replicab* or repeated).ti,ab. |
| #44 | (measure or measures or findings or result or results or test or tests).ti,ab. |
| #45 | 43 and 44 |
| #46 | (generaliza* or generalisa* or concordance).ti,ab. |
| #47 | (intraclass and correlation*).ti,ab. |
| #48 | (discriminative or "known group" or factor analysis or factor analyses or dimension* or subscale*).ti,ab. |
| #49 | (multitrait and scaling and (analysis or analyses)).ti,ab. |
| #50 | 45 or 46 or 47 or 48 or 49 |
| #51 | (item discriminant or interscale correlation* or error or errors or "individual variability").ti,ab. |
| #52 | (variability and (analysis or values)).ti,ab. |
| #53 | (uncertainty and (measurement or measuring)).ti,ab. |
| #54 | ("standard error of measurement" or sensitiv* or responsive*).ti,ab. |
| #55 | ((minimal or minimally or clinical or clinically) and (important or significant or detectable) and (change or difference)).ti,ab. |
| #56 | (small* and (real or detectable) and (change or difference)).ti,ab. |
| #57 | (meaningful change or "ceiling effect" or "floor effect" or "Item response model" or IRT or Rasch or "Differential item functioning" or DIF or "computer adaptive testing" or "item bank" or "cross-cultural equivalence").ti,ab. |
| #58 | 51 or 52 or 53 or 54 or 55 or 56 or 57 |
| #59 | 25 or 37 or 42 or 50 or 58 |

**Table A.4. Embase Search A.**

| 1. Muscular Dystrophy/ |  |
| --- | --- |
| 2. duchenne*.mp. |  |
| 3. 1 or 2 |  |
| 4. (HR-PRO or HRPRO or HRQL or HRQoL or QL or QoL).ti,ab. or quality of life.mp. or (health index* or health indices or health profile*).ti,ab. or health status.mp. or ((patient or self or child or parent or carer or proxy) adj (appraisal* or appraised or report or reported or reporting or rated or rating* or based or assessed or assessment*)).ti,ab. or ((disability or function or functional or functions or subjective or utility or utilities or wellbeing or well being) adj2 (index or indices or instrument or instruments or measure or measures or questionnaire* or profile or profiles or scale or scales or score or scores or status or survey or surveys)).ti,ab. |  |
| 5. 'Pediatric Quality of Life Inventory'.mp. |  |
| 6. PedsQL.mp. |  |
| 7. (SF-36 or EQ-5D*).mp. |  |
| 8. "World Health Organization Quality of Life".mp. |  |
| 9. (KIDSCREEN or Pittsburgh Sleep Quality questionnaire or PSQI).mp. [mp=title, abstract, heading word, table of contents, key concepts, original title, tests & measures] |  |
| 10. "Childrens Assessment of Participation and Enjoyment".mp. |  |
| 11. (CAPE or "Child Health Questionnaire" or CHQ* or Health Utilities Index Questionnaire or HUI*).mp. |  |
| 12. (Fatigue Severity Scale or FSS or "Hospital Anxiety and Depression Scale" or HADS or COPE Inventory or "Quality of Life in Neuromuscular Disease").mp. |  |
| 13. (QoL-NMD DISABKIDS or "Depressionsiventar fur kinder und Jugendliche" or DIKJ or Beck Depression Inventory or BDI or CARE-NMD or State-Trait Anxiety Inventory or STAI or "Life Satisfaction Index for Adolescents" or LSI-A or Quality of Life Evaluation Scale or AUQUEI or Activity Limitations Questionnaire or ACTIVLIM).mp. |  |
| 14. (sf12 or sf 12 or short form 12 or shortform 12 or sf twelve or sftwelve or shortform twelve or short form twelve).mp. |  |
| 15. (sf36 or sf 36 or short form 36 or shortform 36 or sf thirtysix or sf thirty six or shortform thirtysix or shortform thirty six or short form thirtysix or short form thirty six).mp. |  |
| 16. 5 or 6 or 7 or 8 or 9 or 10 or 11 or 12 or 13 or 14 or 15 |  |
| 17. 4 or 16 |  |
| 18. 3 and 17  **Table A.5. Embase Search B.**   \| 1. Duchenne muscular dystrophy/ \|  \| \| --- \| --- \| \| 2. duchenne*.mp. \|  \| \| 3. 1 or 2 \|  \| \| 4. Autoquestionnaire Qualite de vie Enfant Image.mp. \|  \| \| 5. (Behavior Assessment System for Children or BASC or Parent Form 50 or PF50 or DUX-25 or EuroQoL 5-domain or Functional Independence Measure* or FIM).mp. \|  \| \| 6. (WeeFIM or Life Satisfaction Index or LSI or LSIA or Neurological Disorders Quality of Life Questionnaire or NeuroQOL or pediatric NeuroQOL or Offer Self-Image Questionnaire for Adolescents or OSIQ).mp. \|  \| \| 7. (Pediatric Outcomes Data Collection Instrument or PODCI or Neuromuscular module or DMD module or Multidimensional Fatigue Scale or Generic Short-Form or SF15).mp. \|  \| \| 8. (SDQ or 'Strips of Life with Emoticons Questionnaire' or SOLE or 'World Health Organisation Quality of Life Scale-Brief Version' or WHOQOL-BREF).mp. \|  \| \| 9. ('Strength and Difficulties Questionnaire').mp. [mp=title, abstract, heading word, drug trade name, original title, device manufacturer, drug manufacturer, device trade name, keyword, floating subheading word, candidate term word] \|  \| \| 10. "Individualized Neuromuscular Quality of Life Questionnaire".mp. \|  \| \| 11. (INQOL or Child Activity Limitations Interview or CALI or "Satisfaction with Life Scale" or SWLS).mp. \|  \| \| 12. 'Pediatric Quality of Life Inventory'.mp. \|  \| \| 13. ("Pediatric Orthopedic Society of North America Pediatric Musculoskeletal Functional Health Questionnaire" or POSNA or Pittsburgh Sleep Quality Index).mp. \|  \| \| 14. PedsQL.mp. \|  \| \| 15. (SF-36 or EQ-5D*).mp. \|  \| \| 16. "World Health Organization Quality of Life".mp. \|  \| \| 17. (KIDSCREEN or Pittsburgh Sleep Quality questionnaire or PSQI).mp. [mp=title, abstract, heading word, drug trade name, original title, device manufacturer, drug manufacturer, device trade name, keyword, floating subheading word, candidate term word] \|  \| \| 18. "Childrens Assessment of Participation and Enjoyment".mp. \|  \| \| 19. (CAPE or "Child Health Questionnaire" or CHQ* or Health Utilities Index Questionnaire or HUI*).mp. \|  \| \| 20. (Fatigue Severity Scale or FSS or "Hospital Anxiety and Depression Scale" or HADS or COPE Inventory or "Quality of Life in Neuromuscular Disease").mp. \|  \| \| 21. (QoL-NMD DISABKIDS or "Depressionsiventar fur kinder und Jugendliche" or DIKJ or Beck Depression Inventory or BDI or CARE-NMD or State-Trait Anxiety Inventory or STAI or "Life Satisfaction Index for Adolescents" or LSI-A or Quality of Life Evaluation Scale or AUQUEI or Activity Limitations Questionnaire or ACTIVLIM).mp. \|  \| \| 22. (sf12 or sf 12 or short form 12 or shortform 12 or sf twelve or sftwelve or shortform twelve or short form twelve).mp. \|  \| \| 23. (sf36 or sf 36 or short form 36 or shortform 36 or sf thirtysix or sf thirty six or shortform thirtysix or shortform thirty six or short form thirtysix or short form thirty six).mp. \|  \| \| 24. 4 or 5 or 6 or 7 or 8 or 9 or 10 or 11 or 12 or 13 or 14 or 15 or 16 or 17 or 18 or 19 or 20 or 21 or 22 or 23 \|  \| \| 25. 3 and 24 \|  \| \| 26. 25 and COSMIN measurement properties filter (see Table A.3) \|  \|   **Table A.6. PsycINFO Search A.**   \| 1. Muscular Dystrophy/ \|  \| \| --- \| --- \| \| 2. duchenne*.mp. \|  \| \| 3. 1 or 2 \|  \| \| 4. (HR-PRO or HRPRO or HRQL or HRQoL or QL or QoL).ti,ab. or quality of life.mp. or (health index* or health indices or health profile*).ti,ab. or health status.mp. or ((patient or self or child or parent or carer or proxy) adj (appraisal* or appraised or report or reported or reporting or rated or rating* or based or assessed or assessment*)).ti,ab. or ((disability or function or functional or functions or subjective or utility or utilities or wellbeing or well being) adj2 (index or indices or instrument or instruments or measure or measures or questionnaire* or profile or profiles or scale or scales or score or scores or status or survey or surveys)).ti,ab. \|  \| \| 5. 'Pediatric Quality of Life Inventory'.mp. \|  \| \| 6. PedsQL.mp. \|  \| \| 7. (SF-36 or EQ-5D*).mp. \|  \| \| 8. "World Health Organization Quality of Life".mp. \|  \| \| 9. (KIDSCREEN or Pittsburgh Sleep Quality questionnaire or PSQI).mp. [mp=title, abstract, heading word, table of contents, key concepts, original title, tests & measures] \|  \| \| 10. "Childrens Assessment of Participation and Enjoyment".mp. \|  \| \| 11. (CAPE or "Child Health Questionnaire" or CHQ* or Health Utilities Index Questionnaire or HUI*).mp. \|  \| \| 12. (Fatigue Severity Scale or FSS or "Hospital Anxiety and Depression Scale" or HADS or COPE Inventory or "Quality of Life in Neuromuscular Disease").mp. \|  \| \| 13. (QoL-NMD DISABKIDS or "Depressionsiventar fur kinder und Jugendliche" or DIKJ or Beck Depression Inventory or BDI or CARE-NMD or State-Trait Anxiety Inventory or STAI or "Life Satisfaction Index for Adolescents" or LSI-A or Quality of Life Evaluation Scale or AUQUEI or Activity Limitations Questionnaire or ACTIVLIM).mp. \|  \| \| 14. (sf12 or sf 12 or short form 12 or shortform 12 or sf twelve or sftwelve or shortform twelve or short form twelve).mp. \|  \| \| 15. (sf36 or sf 36 or short form 36 or shortform 36 or sf thirtysix or sf thirty six or shortform thirtysix or shortform thirty six or short form thirtysix or short form thirty six).mp. \|  \| \| 16. 5 or 6 or 7 or 8 or 9 or 10 or 11 or 12 or 13 or 14 or 15 \|  \| \| 17. 4 or 16 \|  \| \| 18. 3 and 17 \|  \| |  |

**Table A.7. PsycINFO Search B.**

| 1. Muscular Dystrophy/ |  |
| --- | --- |
| 2. duchenne*.mp. |  |
| 3. 1 or 2 |  |
| 4. Autoquestionnaire Qualite de vie Enfant Image.mp. |  |
| 5. (Behavior Assessment System for Children or BASC or Parent Form 50 or PF50 or DUX-25 or EuroQoL 5-domain or Functional Independence Measure* or FIM).mp. |  |
| 6. (WeeFIM or Life Satisfaction Index or LSI or LSIA or Neurological Disorders Quality of Life Questionnaire or NeuroQOL or pediatric NeuroQOL or Offer Self-Image Questionnaire for Adolescents or OSIQ).mp. |  |
| 7. (Pediatric Outcomes Data Collection Instrument or PODCI or Neuromuscular module or DMD module or Multidimensional Fatigue Scale or Generic Short-Form or SF15).mp. |  |
| 8. (SDQ or 'Strips of Life with Emoticons Questionnaire' or SOLE or 'World Health Organisation Quality of Life Scale-Brief Version' or WHOQOL-BREF).mp. |  |
| 9. ('Strength and Difficulties Questionnaire').mp. [mp=title, abstract, heading word, table of contents, key concepts, original title, tests & measures] |  |
| 10. "Individualized Neuromuscular Quality of Life Questionnaire".mp. |  |
| 11. (INQOL or Child Activity Limitations Interview or CALI or "Satisfaction with Life Scale" or SWLS).mp. |  |
| 12. 'Pediatric Quality of Life Inventory'.mp. |  |
| 13. ("Pediatric Orthopedic Society of North America Pediatric Musculoskeletal Functional Health Questionnaire" or POSNA or Pittsburgh Sleep Quality Index).mp. |  |
| 14. PedsQL.mp. |  |
| 15. (SF-36 or EQ-5D*).mp. |  |
| 16. "World Health Organization Quality of Life".mp. |  |
| 17. (KIDSCREEN or Pittsburgh Sleep Quality questionnaire or PSQI).mp. [mp=title, abstract, heading word, table of contents, key concepts, original title, tests & measures] |  |
| 18. "Childrens Assessment of Participation and Enjoyment".mp. |  |
| 19. (CAPE or "Child Health Questionnaire" or CHQ* or Health Utilities Index Questionnaire or HUI*).mp. |  |
| 20. (Fatigue Severity Scale or FSS or "Hospital Anxiety and Depression Scale" or HADS or COPE Inventory or "Quality of Life in Neuromuscular Disease").mp. |  |
| 21. (QoL-NMD DISABKIDS or "Depressionsiventar fur kinder und Jugendliche" or DIKJ or Beck Depression Inventory or BDI or CARE-NMD or State-Trait Anxiety Inventory or STAI or "Life Satisfaction Index for Adolescents" or LSI-A or Quality of Life Evaluation Scale or AUQUEI or Activity Limitations Questionnaire or ACTIVLIM).mp. |  |
| 22. (sf12 or sf 12 or short form 12 or shortform 12 or sf twelve or sftwelve or shortform twelve or short form twelve).mp. |  |
| 23. (sf36 or sf 36 or short form 36 or shortform 36 or sf thirtysix or sf thirty six or shortform thirtysix or shortform thirty six or short form thirtysix or short form thirty six).mp. |  |
| 24. 4 or 5 or 6 or 7 or 8 or 9 or 10 or 11 or 12 or 13 or 14 or 15 or 16 or 17 or 18 or 19 or 20 or 21 or 22 or 23 |  |
| 25. 3 and 24 |  |
| 26. 25 and COSMIN measurement properties filter (see Table A.3) |  |

**Table A.8. Cochrane Library Search A.**

ID Search

#1 MeSH descriptor: [Muscular Dystrophy, Duchenne] explode all trees

#2 duchenne*:ti,ab,kw

#3 #1 or #2

#4 (HR-PRO or HRPRO or HRQL or HRQoL or QL or QoL):ti,ab

#5 (quality of life):ti,ab,kw

#6 (health index* or health indices or health profile*):ti,ab,kw

#7 (health status):ti,ab,kw

#8 ((patient or self or child or parent or carer or proxy) near (appraisal* or appraised or report or reported or reporting or rated or rating* or based or assessed or assessment*)):ti,ab

#9 ((disability or function or functional or functions or subjective or utility or utilities or wellbeing or well being) near/2 (index or indices or instrument or instruments or measure or measures or questionnaire* or profile or profiles or scale or scales or score or scores or status or survey or surveys)) .ti,ab

#10 #4 or #5 or #6 or #7 or #8 or #9

#11 ("Pediatric Quality of Life Inventory" or PedsQL or SF-36 or EQ-5D* or "World Health Organization Quality of Life" or WHOQoL or KIDSCREEN or Pittsburgh Sleep Quality questionnaire or PSQI or "Children's Assessment of Participation and Enjoyment" or CAPE or "Child Health Questionnaire" or CHQ* or Health Utilities Index Questionnaire or HUI* or Fatigue Severity Scale or FSS or "Hospital Anxiety and Depression Scale" or HADS or COPE Inventory or "Quality of Life in Neuromuscular Disease" or QoL-NMD DISABKIDS or "Depressionsiventar fur kinder und Jugendliche" or DIKJ or Beck Depression Inventory or BDI or CARE-NMD or State-Trait Anxiety Inventory or STAI or "Life Satisfaction Index for Adolescents" or LSI-A or Quality of Life Evaluation Scale or AUQUEI or Activity Limitations Questionnaire or ACTIVLIM):ti,ab,kw

#12 (sf12 or sf 12 or short form 12 or shortform 12 or sf twelve or sftwelve or shortform twelve or short form twelve):ti,ab,kw

#13 (sf36 or sf 36 or short form 36 or shortform 36 or sf thirtysix or sf thirty six or shortform thirtysix or shortform thirty six or short form thirtysix or short form thirty six):ti,ab,kw

#14 #11 or #12 or #13

#15 #10 or #14

#16 #3 and #15

**Table A.9. Cochrane Library Search B.**

ID Search

#1 MeSH descriptor: [Muscular Dystrophy, Duchenne] this term only

#2 (duchenne*):ti,ab,kw

#3 #1 or #2

#4 (Autoquestionnaire Qualite de vie Enfant Image):ti,ab,kw

#5 ("Behavior Assessment System for Children" ):ti,ab,kw

#6 (BASC):ti,ab,kw

#7 (Parent Form 50):ti,ab,kw

#8 (PF50 or DUX-25):ti,ab,kw

#9 ("EuroQoL 5-domain"):ti,ab,kw

#10 (Functional Independence Measure* or FIM):ti,kw,ab

#11 (WeeFIM or Life Satisfaction Index or LSI or LSIA or Neurological Disorders Quality of Life Questionnaire or NeuroQOL or pediatric NeuroQOL or Offer Self-Image Questionnaire for Adolescents or OSIQ):ti,ab,kw

#12 (Pediatric Outcomes Data Collection Instrument or PODCI or Neuromuscular module or DMD module or Multidimensional Fatigue Scale or Generic Short-Form or SF15):ti,ab,kw

#13 (SDQ or 'Strips of Life with Emoticons Questionnaire' or SOLE or 'World Health Organisation Quality of Life Scale-Brief Version' or WHOQOL-BREF):ti,ab,kw

#14 ('Strength and Difficulties Questionnaire'):ti,ab,kw

#15 ("Individualized Neuromuscular Quality of Life Questionnaire"):ti,ab,kw

#16 (INQOL or Child Activity Limitations Interview or CALI or "Satisfaction with Life Scale" or SWLS):ti,ab,kw

#17 ('Pediatric Quality of Life Inventory'):ti,ab,kw

#18 ("Pediatric Orthopedic Society of North America Pediatric Musculoskeletal Functional Health Questionnaire" or POSNA or Pittsburgh Sleep Quality Index):ti,ab,kw

#19 (PedsQL):ti,ab,kw

#20 (SF-36 or EQ-5D*):ti,ab,kw

#21 ("World Health Organization Quality of Life"):ti,ab,kw

#22 (KIDSCREEN or Pittsburgh Sleep Quality questionnaire or PSQI):ti,ab,kw

#23 ("Childrens Assessment of Participation and Enjoyment"):ti,ab,kw

#24 (CAPE or "Child Health Questionnaire" or CHQ* or Health Utilities Index Questionnaire or HUI*):ti,ab,kw

#25 (Fatigue Severity Scale or FSS or "Hospital Anxiety and Depression Scale" or HADS or COPE Inventory or "Quality of Life in Neuromuscular Disease"):ti,ab,kw

#26 (QoL-NMD DISABKIDS or "Depressionsiventar fur kinder und Jugendliche" or DIKJ or Beck Depression Inventory or BDI or CARE-NMD or State-Trait Anxiety Inventory or STAI or "Life Satisfaction Index for Adolescents" or LSI-A or Quality of Life Evaluation Scale or AUQUEI or Activity Limitations Questionnaire or ACTIVLIM):ti,ab,kw

#27 (sf12 or sf 12 or short form 12 or shortform 12 or sf twelve or sftwelve or shortform twelve or short form twelve):ti,ab,kw

#28 (sf36 or sf 36 or short form 36 or shortform 36 or sf thirtysix or sf thirty six or shortform thirtysix or shortform thirty six or short form thirtysix or short form thirty six):ti,ab,kw

#29 #4 or #5 or #6 or #7 or #8 or #9 or #10 or #11 or #12 or #13 or #14 or #15 or #16 or #17 or #18 or #19 or #20 or #21 or #22 or #23 or #24 or #25 or #26 or #27 or #28

#30 #3 and #29

#31 "construct validity" or "content validity" or "criterion validity" or "inter rater reliability" or "interrater reliability"

#32 #30 and #31

**Table A.10. CINAHL Search A.**

| **Search ID#** | **Search Terms** | **Search Options** | **Last Run Via** |
| --- | --- | --- | --- |
| S1 | (MH "Muscular Dystrophy, Duchenne") | Search modes - Boolean/Phrase | Interface - EBSCOhost Research Databases  Search Screen - Advanced Search  Database - CINAHL with Full Text |
| S2 | duchenne* | Search modes - Boolean/Phrase | Interface - EBSCOhost Research Databases  Search Screen - Advanced Search  Database - CINAHL with Full Text |
| S3 | S1 OR S2 | Search modes - Boolean/Phrase | Interface - EBSCOhost Research Databases  Search Screen - Advanced Search  Database - CINAHL with Full Text |
| S4 | HR-PRO | Search modes - Boolean/Phrase | Interface - EBSCOhost Research Databases  Search Screen - Advanced Search  Database - CINAHL with Full Text |
| S5 | ((disability or function or functional or functions or subjective or utility or utilities or wellbeing or well being) N2 (index or indices or instrument or instruments or measure or measures or questionnaire* or profile or profiles or scale or scales or score or scores or status or survey or surveys)) | Search modes - Boolean/Phrase | Interface - EBSCOhost Research Databases  Search Screen - Advanced Search  Database - CINAHL with Full Text |
| S6 | HRPRO or HRQL or HRQoL | Search modes - Boolean/Phrase | Interface - EBSCOhost Research Databases  Search Screen - Advanced Search  Database - CINAHL with Full Text |
| S7 | quality of life or health index* or health indices or health profile* or health status | Search modes - Boolean/Phrase | Interface - EBSCOhost Research Databases  Search Screen - Advanced Search  Database - CINAHL with Full Text |
| S8 | TI QL | Search modes - Boolean/Phrase | Interface - EBSCOhost Research Databases  Search Screen - Advanced Search  Database - CINAHL with Full Text |
| S9 | TI QoL | Search modes - Boolean/Phrase | Interface - EBSCOhost Research Databases  Search Screen - Advanced Search  Database - CINAHL with Full Text |
| S10 | ((patient or self or child or parent or carer or proxy) and (appraisal* or appraised or report or reported or reporting or rated or rating* or based or assessed or assessment*)) | Search modes - Boolean/Phrase | Interface - EBSCOhost Research Databases  Search Screen - Advanced Search  Database - CINAHL with Full Text |
| S11 | S4 OR S5 OR S6 OR S7 OR S8 OR S9 OR S10 | Search modes - Boolean/Phrase | Interface - EBSCOhost Research Databases  Search Screen - Advanced Search  Database - CINAHL with Full Text |
| S12 | (Pediatric Quality of Life Inventory or PedsQL or SF-36 or EQ-5D* or World Health Organization Quality of Life or WHOQoL or KIDSCREEN or Pittsburgh Sleep Quality questionnaire or PSQI or Childrens Assessment of Participation and Enjoyment or CAPE or Child Health Questionnaire or CHQ* or Health Utilities Index Questionnaire or HUI* or Fatigue Severity Scale or FSS or Hospital Anxiety and Depression Scale or HADS or COPE Inventory or Quality of Life in Neuromuscular Disease or QoL-NMD DISABKIDS or Depressionsiventar fur kinder und Jugendliche or DIKJ or Beck Depression Inventory or BDI or CARE-NMD or State-Trait Anxiety Inventory or STAI or Life Satisfaction Index for Adolescents or LSI-A or Quality of Life Evaluation Scale or AUQUEI or Activity Limitations Questionnaire or ACTIVLIM) | Search modes - Boolean/Phrase | Interface - EBSCOhost Research Databases  Search Screen - Advanced Search  Database - CINAHL with Full Text |
| S13 | (sf12 or sf 12 or short form 12 or shortform 12 or sf twelve or sftwelve or shortform twelve or short form twelve) | Search modes - Boolean/Phrase | Interface - EBSCOhost Research Databases  Search Screen - Advanced Search  Database - CINAHL with Full Text |
| S14 | (sf36 or sf 36 or short form 36 or shortform 36 or sf thirtysix or sf thirty six or shortform thirtysix or shortform thirty six or short form thirtysix or short form thirty six) | Search modes - Boolean/Phrase | Interface - EBSCOhost Research Databases  Search Screen - Advanced Search  Database - CINAHL with Full Text |
| S15 | S12 OR S13 OR S14 | Search modes - Boolean/Phrase | Interface - EBSCOhost Research Databases  Search Screen - Advanced Search  Database - CINAHL with Full Text |
| S16 | S11 OR S15 | Search modes - Boolean/Phrase | Interface - EBSCOhost Research Databases  Search Screen - Advanced Search  Database - CINAHL with Full Text |
| S17 | S3 AND S16 | Search modes - Boolean/Phrase | Interface - EBSCOhost Research Databases  Search Screen - Advanced Search  Database - CINAHL with Full Text |

**Table A.11. CINAHL Search B.**

| **Search ID#** | **Search Terms** | **Search Options** | **Last Run Via** |
| --- | --- | --- | --- |
| S1 | (MH "Muscular Dystrophy, Duchenne") | Search modes - Boolean/Phrase | Interface - EBSCOhost Research Databases  Search Screen - Advanced Search  Database - CINAHL with Full Text |
| S2 | duchenne* | Search modes - Boolean/Phrase | Interface - EBSCOhost Research Databases  Search Screen - Advanced Search  Database - CINAHL with Full Text |
| S3 | S1 OR S2 | Search modes - Boolean/Phrase | Interface - EBSCOhost Research Databases  Search Screen - Advanced Search  Database - CINAHL with Full Text |
| S4 | Autoquestionnaire Qualite de vie Enfant Image | Search modes - Boolean/Phrase | Interface - EBSCOhost Research Databases  Search Screen - Advanced Search  Database - CINAHL with Full Text |
| S5 | (Behavior Assessment System for Children or BASC or Parent Form 50 or PF50 or DUX-25 or EuroQoL 5-domain or Functional Independence Measure* or FIM) | Search modes - Boolean/Phrase | Interface - EBSCOhost Research Databases  Search Screen - Advanced Search  Database - CINAHL with Full Text |
| S6 | (WeeFIM or Life Satisfaction Index or LSI or LSIA or Neurological Disorders Quality of Life Questionnaire or NeuroQOL or pediatric NeuroQOL or Offer Self-Image Questionnaire for Adolescents or OSIQ) | Search modes - Boolean/Phrase | Interface - EBSCOhost Research Databases  Search Screen - Advanced Search  Database - CINAHL with Full Text |
| S7 | (Pediatric Outcomes Data Collection Instrument or PODCI or Neuromuscular module or DMD module or Multidimensional Fatigue Scale or Generic Short-Form or SF15) | Search modes - Boolean/Phrase | Interface - EBSCOhost Research Databases  Search Screen - Advanced Search  Database - CINAHL with Full Text |
| S8 | (SDQ or 'Strips of Life with Emoticons Questionnaire' or SOLE or 'World Health Organisation Quality of Life Scale-Brief Version' or WHOQOL-BREF) | Search modes - Boolean/Phrase | Interface - EBSCOhost Research Databases  Search Screen - Advanced Search  Database - CINAHL with Full Text |
| S9 | ('Strength and Difficulties Questionnaire') | Search modes - Boolean/Phrase | Interface - EBSCOhost Research Databases  Search Screen - Advanced Search  Database - CINAHL with Full Text |
| S10 | "Individualized Neuromuscular Quality of Life Questionnaire" | Search modes - Boolean/Phrase | Interface - EBSCOhost Research Databases  Search Screen - Advanced Search  Database - CINAHL with Full Text |
| S11 | (INQOL or Child Activity Limitations Interview or CALI or "Satisfaction with Life Scale" or SWLS) | Search modes - Boolean/Phrase | Interface - EBSCOhost Research Databases  Search Screen - Advanced Search  Database - CINAHL with Full Text |
| S12 | 'Pediatric Quality of Life Inventory' | Search modes - Boolean/Phrase | Interface - EBSCOhost Research Databases  Search Screen - Advanced Search  Database - CINAHL with Full Text |
| S13 | ("Pediatric Orthopedic Society of North America Pediatric Musculoskeletal Functional Health Questionnaire" or POSNA or Pittsburgh Sleep Quality Index) | Search modes - Boolean/Phrase | Interface - EBSCOhost Research Databases  Search Screen - Advanced Search  Database - CINAHL with Full Text |
| S14 | PedsQL | Search modes - Boolean/Phrase | Interface - EBSCOhost Research Databases  Search Screen - Advanced Search  Database - CINAHL with Full Text |
| S15 | (SF-36 or EQ-5D*) | Search modes - Boolean/Phrase | Interface - EBSCOhost Research Databases  Search Screen - Advanced Search  Database - CINAHL with Full Text |
| S16 | "World Health Organization Quality of Life" | Search modes - Boolean/Phrase | Interface - EBSCOhost Research Databases  Search Screen - Advanced Search  Database - CINAHL with Full Text |
| S17 | (KIDSCREEN or Pittsburgh Sleep Quality questionnaire or PSQI) | Search modes - Boolean/Phrase | Interface - EBSCOhost Research Databases  Search Screen - Advanced Search  Database - CINAHL with Full Text |
| S18 | "Childrens Assessment of Participation and Enjoyment" | Search modes - Boolean/Phrase | Interface - EBSCOhost Research Databases  Search Screen - Advanced Search  Database - CINAHL with Full Text |
| S19 | (CAPE or "Child Health Questionnaire" or CHQ* or Health Utilities Index Questionnaire or HUI*) | Search modes - Boolean/Phrase | Interface - EBSCOhost Research Databases  Search Screen - Advanced Search  Database - CINAHL with Full Text |
| S20 | (Fatigue Severity Scale or FSS or "Hospital Anxiety and Depression Scale" or HADS or COPE Inventory or "Quality of Life in Neuromuscular Disease") | Search modes - Boolean/Phrase | Interface - EBSCOhost Research Databases  Search Screen - Advanced Search  Database - CINAHL with Full Text |
| S21 | (QoL-NMD DISABKIDS or "Depressionsiventar fur kinder und Jugendliche" or DIKJ or Beck Depression Inventory or BDI or CARE-NMD or State-Trait Anxiety Inventory or STAI or "Life Satisfaction Index for Adolescents" or LSI-A or Quality of Life Evaluation Scale or AUQUEI or Activity Limitations Questionnaire or ACTIVLIM) | Search modes - Boolean/Phrase | Interface - EBSCOhost Research Databases  Search Screen - Advanced Search  Database - CINAHL with Full Text |
| S22 | (sf12 or sf 12 or short form 12 or shortform 12 or sf twelve or sftwelve or shortform twelve or short form twelve) | Search modes - Boolean/Phrase | Interface - EBSCOhost Research Databases  Search Screen - Advanced Search  Database - CINAHL with Full Text |
| S23 | (sf36 or sf 36 or short form 36 or shortform 36 or sf thirtysix or sf thirty six or shortform thirtysix or shortform thirty six or short form thirtysix or short form thirty six) | Search modes - Boolean/Phrase | Interface - EBSCOhost Research Databases  Search Screen - Advanced Search  Database - CINAHL with Full Text |
| S24 | S4 OR S5 OR S6 OR S7 OR S8 OR S9 OR S10 OR S11 OR S12 OR S13 OR S14 OR S15 OR S16 OR S17 OR S18 OR S19 OR S20 OR S21 OR S22 OR S23 | Search modes - Boolean/Phrase | Interface - EBSCOhost Research Databases  Search Screen - Advanced Search  Database - CINAHL with Full Text |
| S25 | S3 AND S24 | Search modes - Boolean/Phrase | Interface - EBSCOhost Research Databases  Search Screen - Advanced Search  Database - CINAHL with Full Text |
| S26 | TX "construct validity" or "content validity" or "criterion validity" or "inter rater reliability" or "interrater reliability" | Search modes - Boolean/Phrase | Interface - EBSCOhost Research Databases  Search Screen - Advanced Search  Database - CINAHL with Full Text |
| S27 | S25 AND S26 | Search modes - Boolean/Phrase | Interface - EBSCOhost Research Databases  Search Screen - Advanced Search  Database - CINAHL with Full Text |
