# Supplementary material for: Measuring quality of life in Duchenne muscular dystrophy: a systematic review of the content and structural validity of commonly used instruments
Source: Health Qual Life Outcomes. 2020 Aug 3;18:263. doi: 10.1186/s12955-020-01511-z (PMC7397669; doi:10.1186/s12955-020-01511-z)
Supplement: Supplementary file 3 — Additional file 3. Quality assurance of the review. Quality assessment of the systematic review against COSMIN guidance. [file 12955_2020_1511_MOESM3_ESM.docx]

**Additional File 2: Quality assurance of the review**

**Table B.1**. Quality assessment of this systematic review against COSMIN guidance.

| **COSMIN criteria** | **Review meets criteria** |
| --- | --- |
| Elements included in the research aim: |  |
| Construct of interest | + |
| Population of interest | + |
| Type of measurement instrument of interest | + |
| Measurement properties of interest | + |
| All available instruments included | ± |
| Only instruments included that have at least some evidence of measurement properties | + |
| Search strategy described | + |
| No search terms or validated search filter used for: |  |
| Measurement properties | − |
| Type of instrument | − |
| Number of databases searched: | 5 |
| Search in at least 2 databases | + |
| MEDLINE/PubMed | + |
| EMBASE | + |
| Additional databases | + |
| Reference checking used | ± |
| No time limits used or good arguments for a time limit | + |
| No language restrictions used | + |
| Inclusion and exclusion criteria clearly described | + |
| Reasons for excluding articles reported | + |
| Abstract selection by at least 2 reviewers? | + |
| Full-text article selection by at least 2 reviewers? | + |
| Abstract and full-text article selection by at least 2 reviewers? | + |
| Methodological quality of studies assessed | + |
| Quality assessment of studies done by at least 2 reviewers | + |
| Data on measurement properties extracted by at least 2 reviewers | + |
| Quality of the instrument (measurement properties) assessed | + |
| Quality assessment of the instrument by at least 2 reviewers | + |
| Results from multiple studies on the same instrument somehow combined (e.g., best evidence synthesis or pooling) | + |
| Data synthesis was performed: |  |
| Per measurement property | + |
| Only for domains (reliability, validity, responsiveness) |  |
| Only for the whole instrument |  |
| Recommendation provided for the best instrument: |  |
| One instrument is recommended per construct | + |
| More instruments are recommended per construct |  |
| No recommendation for the best instrument |  |
| Results for the measurement properties reported as raw data | + |
| Number of measurement properties reported | 2 |
| Conflict of interest or funding source declared | + |
| One of the authors of the review is also the developer of one of the instruments evaluated in the review | − |

+ = meets criteria; − = does not meet criteria; ± = partially meets criteria.
